# Ion-Mediated Carbon Microdomain Engineering Boosting Enhanced Plateau Capacity of Carbon Anode under High Rate Towards High-Performance Sodium Dual-Ion Batteries

**DOI:** 10.1007/s40820-025-02008-4

**Published:** 2026-01-05

**Authors:** Bin Tang, Yuchen Zhang, Bifa Ji, Geng Yu, Yongping Zheng, Xiaolong Zhou, Nuntaporn Kamonsutthipaijit, Pornsuwan Buangam, Sarayut Tunmee, Hideki Nakajima, Ukit Rittihong, Qingguang Pan, Fan Zhang, Yongbing Tang

**Affiliations:** 1 Advanced Energy Storage Technology Research Center, Shenzhen Institutes of Advanced Technology, Chinese Academy of Sciences, Shenzhen, 518055 People’s Republic of China; 2 University of Chinese Academy of Sciences, Beijing, 100049 People’s Republic of China; 3 Synchrotron Light Research Institute (Public Organization), 111 University Avenue, Muang District, Nakhon Ratchasima, 30000 Thailand; 4 Southern University of Science and Technology, Shenzhen, 518055 People’s Republic of China; 5 Guangdong Provincial Key Laboratory of New Energy Materials Service Safety, College of Materials Science and Engineering, Shenzhen University, Shenzhen, People’s Republic of China

**Keywords:** Carbon microdomain engineering, Ion-mediation, Hard carbon anode, Sodium-ion battery, Dual ion battery

## Abstract

Sodium-based dual-ion batteries (SDIBs) have been attracting increasing attention in recent years owing to their low cost, environmental benignancy, and high operating voltage. However, the sluggish ion kinetics of conventional carbon anodes that cannot match the fast capacitive anion intercalation behavior of graphite cathodes constraints on improving power density of SDIBs. Herein, we present an ingenious carbon microdomain engineering strategy to fabricate high-performance carbon anode with ion-mediated high-activity nitrogen species and molecular-scale closed-pore architectures. Experimental characterizations and theoretical investigations demonstrate that Zn^2+^-mediated structural engineering tailors oxidized nitrogen species, which proficiently accelerate the sodium-ion desolvation kinetics; meanwhile the acetate-mediated pore-forming process modulates closed pores, which synergistically afford abundant sodium storage sites for high plateau-region capacity. As a result, the optimized microdomain engineered carbon material (MEC_3_) tailored with the optimal amount of zinc acetate demonstrates an outstanding plateau-region capacity of 253 mAh g^− 1^ even at 1 C, among the highest reported values. Consequently, the MEC_3_||expanded graphite dual-ion battery exhibits an unprecedented cycling stability at high current rate, maintaining 80.6% capacity retention after 10,000 cycles at 10 C, among the best reports. This microdomain engineering strategy provides a new design principle for overcoming kinetic limitations of carbonaceous materials in plateau-dominated sodium storage systems.

**Supplementary Information:**

The online version contains supplementary material available at 10.1007/s40820-025-02008-4.

## Highlights

- Carbon microdomain engineering using ion-mediated structural control tailors oriented high-activity nitrogen species and creates specific closed pores.
- This strategy accelerates sodium-ion desolvation kinetics, thereby enhancing sodium storage performance even at high current densities.
- The optimized carbon material achieves exceptional rate performance and cycling stability, making it one of the top-tier materials for sodium-ion batteries.

**Supplementary Information:**

The online version contains supplementary material available at 10.1007/s40820-025-02008-4.

## Graphical abstract

**Figure:**
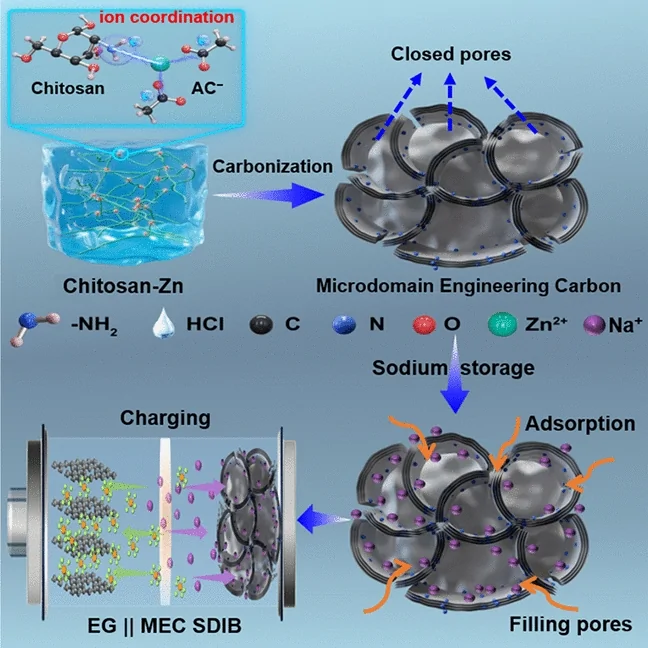

## Introduction

Although lithium-ion batteries (LIBs) are widely used in portable electronics and electric vehicles, the rising cost and limited resources of lithium hinder the broader and long-term application especially in the grid-scale energy storage fields [1–3]. As a more economical and abundant option, sodium-ion based energy storage devices are considered as promising candidates for large-scale energy storage [4, 5]. Among them, sodium-based dual-ion batteries (SDIBs), which usually employ graphite cathodes and carbon anodes, hold significant promise owing to their merits of low cost, environmental benignancy, and high working voltage [6, 7]. However, the sluggish ion kinetics of common carbon anodes cannot satisfy the rapid capacitive anion intercalation behavior of graphite cathodes [8, 9]. Additionally, their sodium storage capacity is relatively low due to the limited active sites [10]. These limitations restrict the achievable power density and energy density of SDIBs. Therefore, developing appropriate anode materials that simultaneously offer high capacity and fast rate capability is imperative to push forward the practical application of SDIBs [11].

Among the various anode materials including graphite [12, 13], amorphous carbon [14], nano-carbon [15], transition metal-based materials [16, 17], alloy materials [18, 19], and organic materials [20], hard carbon materials are the most promising and have been commercially applied anodes owing to their advantages of abundant precursor sources, short-range order graphene-like layers, tunable pore structures, enlarged interlayer spacing, etc. The electrochemical sodium storage behavior in hard carbon materials typically exhibits two distinct voltage-dependent regions: (i) a low-voltage plateau region (< 0.15 V vs. Na^+^/Na), where capacity originates primarily from sodium metal filling of carbon closed pores and (ii) a sloping voltage region (> 0.15 V), where capacity derives from Na^+^ intercalation between graphene-like layers or surface adsorption at defect sites [21]. Recent research efforts have concentrated on promoting the sodium storage capacity of carbonaceous materials by increasing the number of closed pores [22]. However, due to the sluggish sodium storage kinetics in the plateau region, the capacity of carbonaceous anodes significantly decays at high current densities. This makes it challenging to achieve a balance between high specific capacity and good rate performance [23].

It is noteworthy that at high current densities, the main capacity contribution for carbonaceous anodes originates from the slope area with a serious decay in the plateau capacity. The plateau capacity, characterized by its low charge–discharge potential and stable voltage, plays a pivotal role in elevating energy density and ensuring a consistent voltage output of the full battery [24, 25]. To elevate the plateau capacity, various strategies have been employed, involving interlayer spacing regulation of graphene-like layers [26–28] and pore structure design [29, 30]. In terms of pore regulation, typical approaches include conversion of open pores into closed ones [31], construction of graphene-like layers [32], precursor or heat-treatment control [33, 34], template or etching methods [35–37], catalytic or heteroatom-induced pore formation [38], etc. While increasing closed pores can enhance the plateau capacity to some extent, it still declines markedly at high current rates, making it difficult for existing strategies to balance capacity and rate performance (Table S1). Thus, precise microdomain engineering of carbon to simultaneously boost plateau capacity and optimize sodium storage kinetics is essential for advancing high-performance carbon anodes.

Herein, we propose an innovative carbon microdomain engineering strategy employing Zn^2+^ ions as coordination species and acetate as the pore-forming agent to precisely control closed-pore structures at the molecular scale, synergistically combined with a high-activity defect construction, to synthetically enhance the specific capacity and rate performance of carbonaceous materials. Specifically, Zn^2+^-mediated structural engineering constructs the high-activity nitrogen species within the micrographene domains and creates specific closed pores with adequate size and quantity, facilitated by acetate as a pore-forming agent. Experiments and theoretical simulations elucidate that the engineered oxidized nitrogen efficiently accelerates the sodium-ion desolvation, thereby enhancing sodium storage kinetics within the closed pores, which synergistically provide abundant sodium storage sites, contributing to high plateau capacity. This strategy effectively achieves a balance between rate capability and plateau capacity. Consequently, the optimized microdomain engineered carbon (MEC_3_) anode achieves a reversible capacity of up to 427 mAh g^− 1^ at 0.1 C and a high plateau-specific capacity of 253 mAh g^− 1^ even at 1 C, among the best results of the reported carbon anodes. Simultaneously, the SDIB based on the MEC_3_ anode and expanded graphite (EG) cathode delivers outstanding ultralong-term cycling stability and superior rate capability with capacity retention of 80.6% after 10,000 cycles at 10 C, which represents best performance among the SDIB systems.

## Experimental Section

### Raw Materials

Zinc acetate and sodium metal were purchased from Aladdin Reagent Network. Chitosan (medium viscosity, 200–400 mPa s, degree of deacetylation 80% ~ 100%) was purchased from Aladdin. Glass fiber separator (Whatman), Celgard 3501 separator, expanded graphite (EG), conductive carbon black (Super P), Polyvinylidene fluoride (PVDF), N-methyl-2pyrrolidone (NMP), carbon-coated Al, and Al current collectors were purchased from Shenzhen Kejingstar Technology Ltd. Sodium hexafluorophosphate (NaPF_6_), sodium perchlorate (NaClO_4_), ethylene carbonate (EC), dimethyl carbonate (DMC), and ethyl methyl carbonate (EMC) were purchased from Dodochem reagent network. 1 M NaClO_4_ in EC: DMC (1:1, v/v) and 1 M NaPF_6_ in EC: DMC: EMC (1:1:1, v/v/v) were prepared as the electrolytes. All materials were used as purchased unless otherwise indicated.

### Synthesis of MEC Products

Firstly, 15 mL of 0.25 mol L^− 1^ Zn(AC)_2_·2H_2_O solution was prepared. 1.6 g of chitosan was dispersed in 30 mL of deionized water and stirred for 120 min. Then, 6.67 mL of Zn(AC)_2_·2H_2_O solution (0.25 mol L^− 1^) was added. The mixed solution was heated in a water bath at 70 °C for 120 min and then cooled. During the magnetic stirring, 37 wt% concentrated HCl solution (n_(H_^+^_)_: n_(Zn_^2+^_)_ = 1: 1) was added drop by drop and stirred overnight. The uniformly mixed composite system is sticky. The composite carbon precursor CS@Zn(AC)_2_ was obtained by rapid freezing in liquid nitrogen and freeze-drying at − 40 °C for 48 h until complete dryness. The CS@Zn(AC)_2_ was placed in a porcelain boat, heated to 700 °C at a rate of 5 °C min^− 1^ in an argon atmosphere and pre-carbonized for 5 h. The pre-carbonized product was heated to 1300 °C at a rate of 5 °C min^− 1^ in argon atmosphere and calcined at 1300 °C for 120 min. After the heat preservation, it was cooled to 700 °C at a rate of 5 °C min^− 1^, and then cooled naturally with a cooling rate of ~ 1 °C min^− 1^ to obtain the sample MEC_3_. Under the same experimental conditions, MEC_1_, MEC_2_, and MEC_4_ can be prepared by changing the amount of 0.25 mol L^− 1^ of Zn(AC)_2_·2H_2_O solution to 3.33, 5, and 20 mL, respectively, and MEC_0_ can be obtained without adding Zn(AC)_2_·2H_2_O solution.

### Material Characterization

Morphology of materials was characterized by field emission scanning electron microscopy (FE-SEM, Phenom ProX, TESCAN MIRA LMS) and field emission transmission electron microscopy (FE-TEM, FEI Tecnai G2 F20). The corresponding elemental analysis was performed with an energy-dispersive X-ray spectrometer (EDS, 721–01375-00). Near edge X-ray absorption fine structure characterization (NEXAFS) was carried out at the BL3.2Ua: PES of the Synchrotron Light Research Institute (SLRI) (Public Organization), Nakhon Ratchasima, Thailand. The carbon K-edge NEXAFS spectra were measured in the energy range of 275–330 eV at an energy step of 0.1 eV by a partial electron yield mode. X-ray diffraction (XRD, MiniFlex600, Cu K*α* radiation, 1.54 Å) patterns were test with a scanning speed of 5° min^− 1^. Raman spectroscopy (XploRA PLUS) with 532 nm diode laser excitation was employed with Raman light transmittance of 1%. X-ray photoelectron spectroscopy (XPS, Thermo Scientific K-Alpha) was used to test the valence state and chemical bonds. The resolution in Fourier transform infrared spectroscopy (FTIR, INVENIO-S) was set to 4 cm^− 1^, and the samples and background scanning time were set to 32 s. A multi-channel automatic specific surface area and porosity analyzer with a resolution of 0.5 nm (Micromeritics ASAP 2020) were used to test the specific surface area and pore size. The glow discharge optical emission spectroscopy (GDOES, GD PROFILER2) was used to characterize the elemental content of the bulk phase of the material, and the parameters were set to 200 Pa and 20 W. Based on inductively coupled plasma mass spectrometry (ICP-MS, Agilent 7700), the content of zinc was accurately analyzed at ppm level, and the samples were digested by microwave before testing. The element analysis test (EA) was used to accurately analyze the N content of the samples. Small angle X-ray scattering (SAXS) was carried out based on Thailand Synchrotron light research institute (SLRI-BL1.3 W), and the air background was corrected for each test sample. The radiation source is multipole wiggler with the photon energy range of 6 ~ 9 keV and the photon flux at the sample of ~ 2e^9^ phs s^− 1^ and the energy bandwidth is 1%. The Zn K-edge extended X-ray absorption fine structure (EXAFS) characterization was performed based on SLRI-BL5.2. The transmission mode was selected with an energy resolution of 2 × 10^− 4^, and the sample thickness was adjusted to make the edge step in the range of 0.7 ~ 1. The C K-edge NEXAFS characterization was performed based on SLRI-BL8.

### Electrochemical Tests

The CR2016 coin cell was assembled in an argon-filled glove box (Mikarouna) before testing the electrochemical performance of the samples. The MEC samples, PVDF, and SP were fully ground in a mass ratio of 8:1:1, and uniformly mixed with NMP to prepare the MEC electrodes on a carbon-coated aluminum foil. The loading amount of the active materials after vacuum oven drying is ~ 1 mg cm^− 2^. The sodium metal is used as the counter electrode. The glass fiber (Whatman) was used as the separator, and in the half cell (Na || MEC), 1 M NaClO_4_ in EC/DMC (1:1, v/v) was employed to evaluate the intrinsic Na⁺ storage of MEC. In the dual-ion batteries (MEC || EG), EG, SP, and PVDF were mixed in a mass ratio of 8:1:1, then they were coated on aluminum foil as the cathode with a mass loading of ~ 2 mg cm^− 2^. To support Na⁺ storage in the anode and reversible PF_6_⁻ intercalation in the EG cathode, 1 M NaPF_6_ in EC/DMC/EMC (1:1:1, v/v/v) was selected as the electrolyte. The voltage range of DIB operation was 2.0–4.8 V. Before assembling the DIBs, the MEC_3_ anode was first discharged to 0.0001 V in the half cell for activation to promote the in situ formation of a stable solid electrolyte interphase (SEI) film on the MEC_3_ surface, and to pre-compensate for the initial irreversible capacity loss in the DIBs. This activation process could improve the initial Coulombic efficiency, enhance cycling stability, and prolong the battery lifespan. The galvanostatic charge–discharge tests and cycled charge–discharge tests were carried out at 25 °C using a Neware battery testing system (CT4008A). Cyclic voltammetry (CV) curves and electrochemical impedance spectral (EIS) measurements were tested on Princeton electrochemical workstation (VersaSTAT).

### DFT Simulations

All computational simulations detailed herein were executed utilizing the Vienna ab initio simulation package (VASP), employing spin-polarized density functional theory (DFT) with the projector-augmented wave (PAW) method and a plane-wave cutoff energy set at 450 eV. The exchange–correlation energy was treated within the framework of the generalized-gradient approximation (GGA), utilizing the Perdew-Burke-Ernzerhof (PBE) functional. The thresholds for energy and force convergence were set as 1 × 10^− 5^ eV and 0.01 eV Å^− 1^, respectively. The supercell of the models was constructed with lattice parameters of 30 × 30 × 30 Å^3^, and k-space sampling employed a Monkhorst–Pack grid using a 1 × 1 × 1 k-point mesh, corresponding to a k-spacing of < 0.035 Å^− 1^. The Arrhenius equation was employed to determine the desolvation rate of solvated ions at certain energy and temperature. Within the stepwise desolvation model, the desolvation energy for detaching the *n*-th (*n* = 1, 2, 3, 4, 5, 6) EC solvent moiety from the sodium ion is expressed as:

1 $$E_{{{\mathrm{desolv}}}}^{\left( n \right)} = E_{{{\mathrm{tot}}}} \left[ {{\mathrm{slab}} + {\mathrm{Na}}^{ + } \left( {{\mathrm{EC}}} \right)_{6 - n} } \right] + E\left[ {{\mathrm{EC}}} \right] - E_{{{\mathrm{tot}}}} \left[ {{\mathrm{slab}} + {\mathrm{Na}}^{ + } \left( {{\mathrm{EC}}} \right)_{7 - n} } \right]$$

where *n* = 6 for the fully coordinated state, *E*_tot_[slab + Na^+^(EC)_7-*n*_] and *E*_tot_[slab + Na^+^(EC)_6-*n*_] denote DFT total energies of the electrode and EC-coordinated Na^+^ system, corresponding to the states immediately before and after detachment of the *n*-th EC solvent molecule, respectively. *E*[EC] is the energy of an isolated EC molecule.

## Results and Discussion

### Carbon Microdomain Engineering and Structural Characterizations

**Fig. 1:**
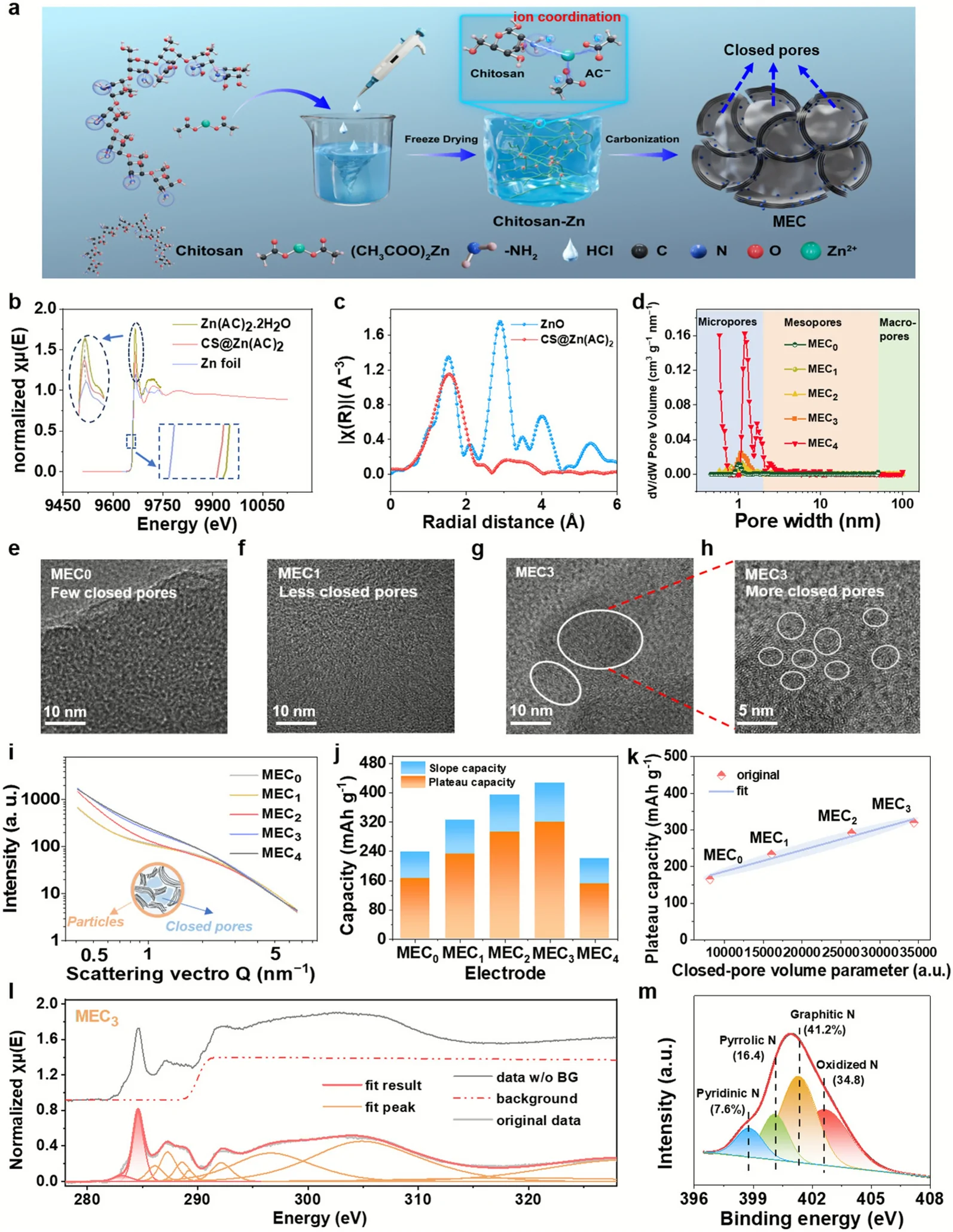
Morphological structural characterizations and atomic structure analysis. **a** Synthesis diagram and mechanism model of CS@Zn(AC)_2_. **b** Zn K-edge EXAFS. **c** Fourier transform EXAFS spectra. **d** Pore diameter distribution curves for MEC_0_ ~ MEC_4_. HR-TEM images of **e** MEC_0_, **f** MEC_1_, and **g-h** MEC_3_. **i** SAXS patterns for MEC_0_ ~ MEC_4_. **j** Capacity comparison of MEC_0_ ~ MEC_4_. **k** Quantitative relationship curve between closed-pore volume parameter and plateau-specific capacity. **l** Carbon K-edge NEXAFS spectra of MEC_3_ in the energy range of 275 ~ 335 eV. **m** XPS spectra of N 1*s* for MEC_3_

Herein, the precursor was prepared through the chemical coordination reaction of cost-effective and biodegradable chitosan with zinc acetate (Zn(AC)_2_) as a functional reagent, named CS@Zn(AC)_2_. Subsequently, the MECs were fabricated after a stepwise carbonization process. The as-obtained carbon samples are denoted as MEC_1_ ~ MEC_4_ by progressively increasing the dosage of Zn(AC)_2_ with a concentration of 0.25 mol L^− 1^ from 3.33 to 5 mL, 6.67 mL, and finally, 20 mL under the same experimental conditions, and the experimental details are listed in the Supporting Information. As a comparison, a baseline sample labeled MEC_0_ was prepared by direct stepwise carbonization of chitosan alone. Chitosan acts as both the precursor and doping source for carbonaceous materials, where its amino functional groups as the Lewis alkaline are activated under acidic conditions and the N atoms ionize the delocalized electrons, which leads to the coordination of Zn^2+^ ions on the activated amino group (Fig. 1a). As revealed by FTIR spectra in Fig. S1, the broad band at 3349 cm^− 1^ ascribed to N − H and H − O stretching vibrations shifts to high frequency (at 3386 cm^− 1^) with increased intensity, and the absorption peak at 1561 cm^− 1^ corresponding to the bending vibration of the amino group is significantly enhanced, indicating a charge redistribution between the chitosan’s amino groups and strong polar Zn^2+^ ions in CS@Zn(AC)_2_ [39, 40]. In addition, the − OH bending vibrations of both phenols and alcohols undergo a shift toward high frequencies with enhanced intensities, attributable to the AC^−^-induced electronic modulation. The atomic-level coordination configuration and bonding analysis were further carried out by EXAFS (Fig. 1b–c). The near absorption spectrum of Zn-K edge in CS@Zn(AC)_2_ exhibits a red shift compared to Zn(AC)_2_, accompanied by an energy level surpassing that of Zn foil, indicating the reduction in the valence state of Zn^2+^ upon interaction with the delocalized electrons within the activated amino group. Furthermore, a prominent Zn − N coordination peak at 1.53 Å stands out in the Fourier transform EXAFS spectra, different from the multiple signals of ZnO, further proving the presence of the functional motifs − C − NH_2_ − Zn^2+^ − (AC^−^)_2_ − within CS@Zn(AC)_2_ [41, 42]. This directional metal–ligand interaction enables molecular-scale architectural control over the carbon precursor, facilitating precise microstructure reconstruction. The minimal concentration fluctuation of O and Zn elements in GDOES, in Fig. S2, indicates uniform distribution of elements in depth. During the subsequent carbonization process, the intricate turbostratic micrographene domain architectures could be formed through synergistic coordination between Zn^2+^-mediated induction and AC^−^ as a gentle nano-porogen agent. After calcination at 1300 °C, the pre-carbonized product is converted into hard carbon, accompanied by a pronounced enhancement of the D and G bands in the Raman spectra (Fig. S3).

N_2_ adsorption–desorption tests were performed on the products MEC_0_ ~ MEC_4_ to analyze their specific surface area and pore distribution, where the specific surface areas of MEC_0_ ~ MEC_3_ are small with MEC_3_ of just 39.13 m^2^ g^− 1^ (Fig. S4, Table S2), dominated by micropores of 0.5 ~ 2 nm (Figs. 1d and S5, Table S2). The excessive presence of Zn(AC)_2_ enhances pore formation, yielding a surge in micropores and mesopores, thereby boosting the specific surface area of MEC_4_ to 229.73 m^2^ g^− 1^. Importantly, N_2_ adsorption is insensitive to the closed pores that are pertinent to sodium storage activity. This trend is verified by high-resolution TEM images (Fig. 1e–h), where the carbon layer of MEC_0_ appears in a distinct disordered state, while the graphene-like layers from MEC_0_ to MEC_3_ show a continuous increase accompanied by a significant rise in the number of closed pores (highlighted by white circles). This phenomenon could be attributed to the synergistic action of Zn^2+^ ions as coordination species and AC^−^ ions as nano-porogen agents. Their interplay facilitates the precise tailoring of carbon microdomain topology through coordinated chemical etching and nitrogen stabilization, which could promote the short-range ordered assembly of graphene-like crystallites, yielding three-dimensional closed-pore networks within the N-enriched carbon microdomains. To further elucidate the closed-pore characteristics, the SAXS was carried out. The contrasting electron cloud densities inside and outside the pores lead to incoherent X-ray scattering, with the scattering intensity peak within the scattering vector range of Q from 0.4 to 6 nm^− 1^. Obviously, the scattering intensity of MEC_3_ is progressively enhanced at the same scattering vector compared to that of MEC_0_, indicating a steady increase in the number of nano-closed pores for MEC_3_ (Fig. 1i and S6). However, the notable scattering intensity within the range of 0.4 ~ 1.0 nm^− 1^ for MEC_4_ is attributed to its abundant mesopores, which are usually assigned to open pores. The above results are consistent with the N_2_ adsorption–desorption results. XRD patterns further confirm this rule (Fig. S7), where the half-peak width of the (002) and (100) plane decreases continuously from MEC_0_ to MEC_3_ (Fig. S8), indicating the enhancement in both the degree of graphitization and c-axis stacking order (Table S3). By finely fitting the scattering peaks of SAXS patterns, we obtain quantitative insight into critical parameters such as the average pore sizes, pore specific surface area parameters, and closed-pore volume parameters (Table S4). With increasing the dosage of Zn(AC)_2_, the pore-forming efficacy intensifies, leading to an increase in the closed-pore volume parameter for MEC_3_. Considered the high proportion of mesopores for MEC_4_, it displays much smaller closed-pore volume parameter than MEC_3_. Charge–discharge test of half cells based on the MEC products shows that the plateau capacity is significantly improved from MEC_0_ (165 mAh g^− 1^) to MEC_3_ (320 mAh g^− 1^) at current rate of 0.1 C (1 C = 300 mA g^−1^, Figs. S9 and 1j). Surprisingly, it is discovered that the plateau capacity demonstrates a linear positive correlation with the closed-pore volume parameter from MEC_0_ to MEC_3_ (Fig. 1k), where the closed-pore volume parameter was derived by fitting the SAXS patterns (see Supporting Information), and the plateau capacities were obtained from galvanostatic charge and discharge tests. In contrast, MEC_4_ undergoes a sharp decline in plateau capacity (151 mAh g^− 1^), ascribed to the abundant open pores. In addition, MEC_3_ also exhibits a favorable initial Coulombic efficiency (~ 93.35%), compared with 87.46%, 90.56%, and 91.13% for MEC_0_, MEC_1_, and MEC_2_, respectively, where this monotonic evolution demonstrates that closed-pore engineering could suppress electrolyte decomposition and substantially improve the reversibility of sodium storage. Meanwhile, the initial Coulombic efficiency of MEC_4_ decreases to ~ 88.5% owing to irreversible side reactions in its open pores (Fig. S10). Considering that, MEC_3_ is selected as the optimal carbon product and used for the following studies.

To further elaborate the structure feature of MEC_3_, comprehensive characterization techniques were conducted. SEM and EDS images display a uniform graphene-like microdomain with the presence of uniform C, N, and O elements in the MEC_3_ (Fig. S11). To in-depth understand the elemental composition of the MEC_3_, ICP-MS and EA test were employed, where the content of N reaches 1.15 wt%, while the content of Zn is exceedingly low of 0.00155 wt%, effectively excluding its potential effect on the properties of the MEC_3_ (Table S5). Furthermore, the high-resolution XPS spectrum of Zn 2*p* (Fig. S12) shows no detectable Zn signal, further confirming the negligible influence of Zn species. Furthermore, the *sp*^2^/(*sp*^2^ + *sp*^3^) ratio in the samples was further quantitatively analyzed by NEXAFS to evaluate the graphitization degree [35, 43]. As depicted in Figs. 1l and S13, the pre-edge resonance located at ~ 284.6 eV is associated with C 1*s* → π*(C = C) transition from *sp*^2^ sites, including the contributions from the π*(C≡C) state [43]. The high energy edge from ~ 288.6 to ~ 327.5 eV is related to C 1*s* → σ*(C–C) transitions from *sp*, *sp*^2^, and *sp*^3^ hybridization, showing the fitting peak in the corresponding energy range [43]. It is found that the proportion of *sp*^2^ hybrid carbon atoms in MEC_0_ (6.36%) is lower than that in MEC_3_ (7.61%), which is attributed to the enhanced formation of graphene-like structures in MEC_3_ induced by Zn^2+^ [44]. Meanwhile, XPS displays the introduction of specialized N heteroatoms within the graphene-like layers. An insightful comparison between conventional N doping in MEC_0_ and efficient N doping in MEC_3_ reveals a fundamental disparity (Figs. 1m, S14, and S15), where the concentration of oxidized N in MEC_3_ is significantly higher than that in MEC_0_, with sharply decrease of graphitic N and negligible change of pyridinic N and pyrrolic N [45], which could be attributed to the orientated coordination effect of Zn^2+^.

### Sodium Storage Performance

**Fig. 2:**
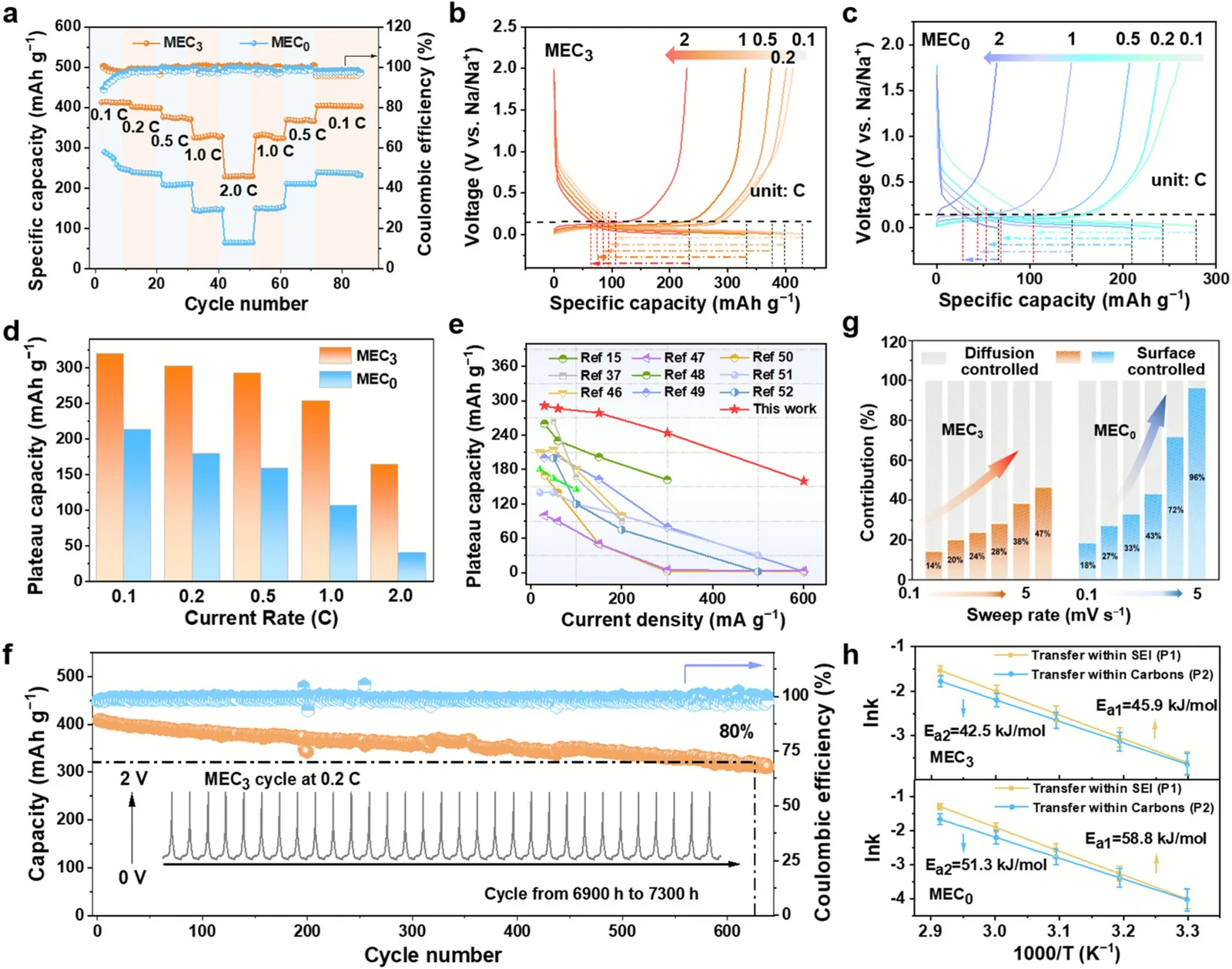
Electrochemical characterizations of Na | | MEC_3_ half cell. **a** Rate capabilities at 0.1 ~ 2 C for MEC_0_ and MEC_3_. GCD curves of **b** Na | | MEC_3_ and **c** Na || MEC_0_ from 0.1 to 2 C. **d** Comparison of plateau-region rate performance of MEC_3_ and MEC_0_. **e** Comparison of the plateau-region rate performance of typical carbon-based anodes reported previously. **f** Cycling performance of MEC_3_ at 0.2 C with an insert of GCD curve from 6900 to 7300 h. **g** Calculated contributions of the surface-controlled and diffusion-controlled proportions varying from 0.1 to 5.0 mV s^− 1^ for MEC_0_ and MEC_3_. **h** Arrhenius plots (ln(k) vs 1/T) for the MEC_0_ and MEC_3_ obtained at different reaction temperatures

The sodium-based half cells with MEC_0_ and MEC_3_ electrode were assembled to evaluate the electrochemical performance of the as-prepared samples in a conventional carbonate electrolyte. As illustrated in Fig. 2a, the MEC_3_ achieves remarkable specific capacities of 427, 396, 375, 325, and 243 mAh g^− 1^ at various current densities of 0.1, 0.2, 0.5, 1, and 2 C, respectively. In contrast, MEC_0_ experiences significant capacity fade at high rates, decaying to only 64 mAh g^− 1^ at 2 C. Notably, MEC_3_ significantly boosts the rate performance in the plateau region, as evidenced by minimal attenuation of plateau capacity from 0.1 to 2 C. As shown in Fig. 2b, MEC_3_ achieves a high plateau capacity of ~ 338 mAh g^− 1^ at 0.1 C, and retains a distinct plateau capacity of ~ 253 mAh g^− 1^ (76% of its total capacity) even at high rate of 1 C. Conversely, the plateau capacity of MEC_0_ diminishes to 105.8 mAh g^− 1^ at 1 C, only 33% of the plateau capacity at 0.2 C (Figs. 2c and S16). As the current rate escalates, the plateau capacity retention of MEC_3_ is significantly higher than those of MEC_0_ (Fig. 2d), and much better than most reported results (Fig. 2e, Table S6) [15, 37, 46–52]. The above results verify that the carbon microdomain engineering design of molecular-scale closed-pore architectures and an efficient defect protocol with rich oxidized N can accelerate the sodium storage kinetics, contributing to excellent rate performance with high plateau capacity at high current rates, therefore achieving a great balance between the rate capability and high plateau capacity. Moreover, the Na||MEC_3_ half cell exhibits good cycling stability with capacity retention of 80% for over 600 cycles at 0.2 C (Fig. 2f), meanwhile the voltage remains stable throughout the long-term charge and discharge processes (insets in Figs. 2f and S17), manifesting great structure robustness. To probe the structural evolution, MEC_3_ after 100 cycles was examined by SEM (Fig. S18) and HR-TEM (Fig. S19). The analyses reveal that the morphology and structure especially for the closed pores remain virtually unchanged, highlighting the remarkable microstructural stability of MEC_3_ upon prolonged cycling.

The electrochemical behavior and reaction kinetics of carbon samples were measured and analyzed by CV measurements in the half-cell configuration from 0.1 to 5.0 mV s^− 1^. At a scan rate of 0.1 mV s^− 1^, MEC_3_ exhibits a pair of sharp redox peaks at 0.11/0.008 V, corresponding to Na filling into nanopores below 0.15 V (insets in Fig. S20a). In contrast, MEC_0_ displays a larger potential shift of redox peaks and polarization, with greater irreversibility in the initial 2 CV cycles, possibly attributed to the proliferation of SEI films (insets in Fig. S20b). Generally, the response current in the slope area primarily stems from the surface-controlled processes, whereas the plateau region is dominated by the diffusion-controlled currents. The contributions of the surface- or diffusion-controlled currents for MEC_3_ and MEC_0_ at different scan rates were calculated using Eq. (2) [53–55]:

2 $$i\left( V \right) = k_{1} v + k_{2} v^{0.5}$$

where ${k}_{1}v$ and ${k}_{2}{v}^{0.5}$ represent the response currents of the surface-controlled reaction and the diffusion-controlled reaction, respectively. As shown in Fig. 2g, the diffusion-controlled reaction predominantly contributes to the capacities of both MEC_3_ (~ 86%) and MEC_0_ (~ 82%) in the plateau region at 0.1 mV s^− 1^. However, the proportion of diffusion-controlled current corresponding to the plateau region in MEC_0_ undergoes a sharp decline, with its contribution plummeting from 82% to merely 4% as the scan rate increases from 0.1 to 5 mV s^− 1^, indicating that the severe degradation of plateau capacity under high current rate. On the contrary, MEC_3_ exhibits a steady decline in diffusion-controlled current contribution with increasing scan rates, while the plateau capacity remains preeminent even at high scan rates, signifying a remarkable enhancement in the kinetics of the plateau region, which profits from the fine carbon microdomain design involving moderately closed pores and efficient N doping with rich oxidized N. Furthermore, galvanostatic intermittent titration technique (GITT) was employed to evaluate the Na⁺ diffusion coefficients (D-Na⁺) of MEC_3_ and MEC_0_. As shown in Figs. S21 and S22, MEC_3_ consistently exhibits markedly higher diffusion coefficient than MEC_0_ during both charging and discharging processes. This finding provides direct evidence that the carbon microdomain engineering strategy effectively enhances Na⁺ diffusion kinetics. To further compare the electrochemical behavior and thermodynamics of the MEC products, EIS technique was employed. The battery develops a stable SEI film after being activated; therefore, the embedded Na^+^ ions in the carbon structure undergo crucial transport processes both within the SEI film (light blue region, P1, in Fig. S23) and through the active material (orange region, P2, in Fig. S23), which is shown as two distinct high-frequency semicircles in the Nyquist plots. It is noteworthy that the fitted parameters show that the series resistance (*R*_s_) of MEC_3_ (2.11 Ω) is lower than that of MEC_0_ (2.67 Ω). More importantly, the charge-transfer resistance within the SEI film (*R*_ct1_) and in the bulk phase (*R*_ct2_) of MEC_3_ (29.45 and 14.66 Ω, respectively) are significantly reduced compared with those of MEC_0_ (56.16 and 70.44 Ω, respectively) (Table S7). This confirms that the synergistic effect of Zn^2+^ ions and acetate ions greatly enhances the electronic conductivity of MEC_3_ and accelerates the charge-transfer kinetics. Moreover, EIS tests were performed from 30 to 70 °C to compare the activation energy of MEC_3_ and MEC_0_ (Fig. S24). The marked decreases in the charge-transfer resistance are observed for both MEC_3_ and MEC_0_. Besides, the activation barrier was calculated by Arrhenius equation [56, 57], which is expressed as:

3 $$lnk = - \frac{{E_{a} }}{RT} + lnA$$

where *A* is the pre-exponential factor, ${E}_{a}$ is the apparent activation barrier, *R* is the ideal gas constant, and *T* is the absolute temperature (Kelvin). The activation energy of sodium ion transferred within the SEI film and within the active material can be obtained by fitting data of two semicircles, respectively. Figure  2h shows that the activation energy required for Na^+^ transport in MEC_3_ is significantly lower than that in MEC_0_ within both the SEI films (45.9 vs. 58.8 kJ mol^− 1^) and the active materials (42.5 vs. 51.3 kJ mol^− 1^), demonstrating the ingenious carbon microdomain engineering strategy can effectively decrease the activation energy and facilitate fast kinetics.

### Sodium Storage Mechanism

**Fig. 3:**
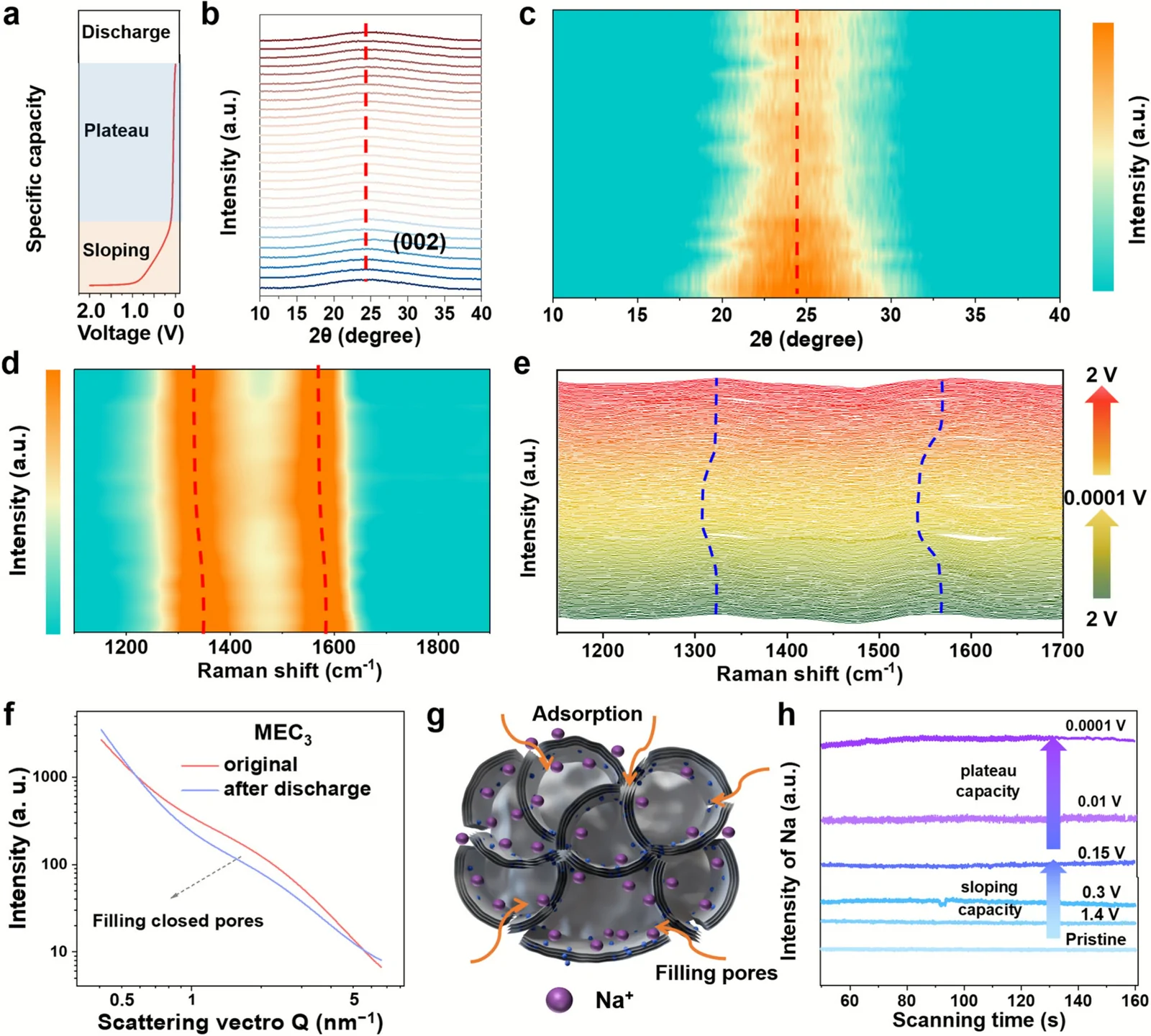
Characterizations of sodium storage mechanism of MEC_3_. **a** Typical discharge curves corresponding to ex situ XRD and Raman characterizations. **b, c** Ex situ XRD spectra during discharging. **d** Ex situ Raman spectra during discharging. **e** In situ Raman spectra during charging and discharging. **f** SAXS patterns before and after discharging. **g** Schematic illustration of sodium-ion storage. **h** Ex situ GDOES spectra of sodium element in MEC_3_ during discharging

The storage behavior of Na^+^ ions within graphene-like interlayer was investigated by XRD technique. During the discharge process (Fig. 3a), the intensity of (002) peak diminishes gradually with negligible shift (Fig. 3b, c), indicating no intercalation of Na^+^ ions into the graphitic carbon interlayers [58, 59]. To further investigate the sodium storage mechanism in the MEC_3_ material and reveal the interaction mechanism between carbon atoms and Na^+^, Raman spectroscopy was employed. As ex situ Raman spectra depicted in Fig. 3d and in situ Raman spectra as shown in Fig. 3e, the intensities of D band and G band remain stable at high voltages, indicating minimal influence on the C–C bonds ascribed to sodium adsorption in the slope region [60]. During the deep discharging process, a red shift in the G band is observed, likely due to the electron transfer to carbon atoms, causing the occupation of the π* anti-bonding orbitals and weakening of the C–C bonds, indicating possible sodium storage through pore-filling mechanism [61, 62]. The variations in the position and intensity of the D band peak during discharging indicate the elimination or restoration of defects as reactive sites within the carbon structure [63]. Above characterizations provide direct spectroscopic evidence for the sequential “adsorption–filling” mechanism. Meanwhile, in situ Raman spectra display the reversed shifts during the charging process, demonstrating high reversibility of Na⁺ storage in MEC_3_. As in situ FTIR spectra depicted in Fig. S25, during the discharge process, the C = O peaks of EC molecules shift to low wavenumber due to their coordination with Na^+^. MEC_3_ displays wider red shift compared to MEC_0_, indicating a deeper desolvation process [15, 63], which confirms that MEC_3_ achieves greater desolvation likely due to the oriented high-activity nitrogen species and creates specific closed pores. Furthermore, both the initial MEC_3_ and MEC_3_ at the completely discharged state (0.0001 V) were characterized by synchrotron radiation light source-SAXS (Fig. 3f). The scattering intensities of MEC_3_ after discharging decrease in the scattering vector range of Q = 0.6 to 5 nm^− 1^, indicating the attenuated contrast of electron cloud density between the interior and exterior of pores, thereby weakening the X-ray scattering effect, which is mainly attributed to the filling of Na^+^ ion into the closed pores (Fig. 3g). Consistently, the pore volume parameter (V) of MEC_3_ after discharging shows a significant reduction, directly confirming the pore-filling effect of sodium ions into hard carbon. To track the Na^+^ content dynamic in MEC_3_ during discharging, GDOES was conducted, where the variation in Na^+^ content under varying discharge states is directly correlated with the capacity ratio (Fig. 3h). Simultaneously, minimal fluctuation in the Na^+^ content along the depth of the MEC_3_ related to the scanning time during discharge, indicating rapid Na^+^ transport kinetics in the carbon matrix. The above characterizations verify that MEC_3_ mainly demonstrates the “adsorption-filling pores” sodium storage mechanism.

### Theoretical Simulations on Sodium Storage Enhancement Mechanism

**Fig. 4:**
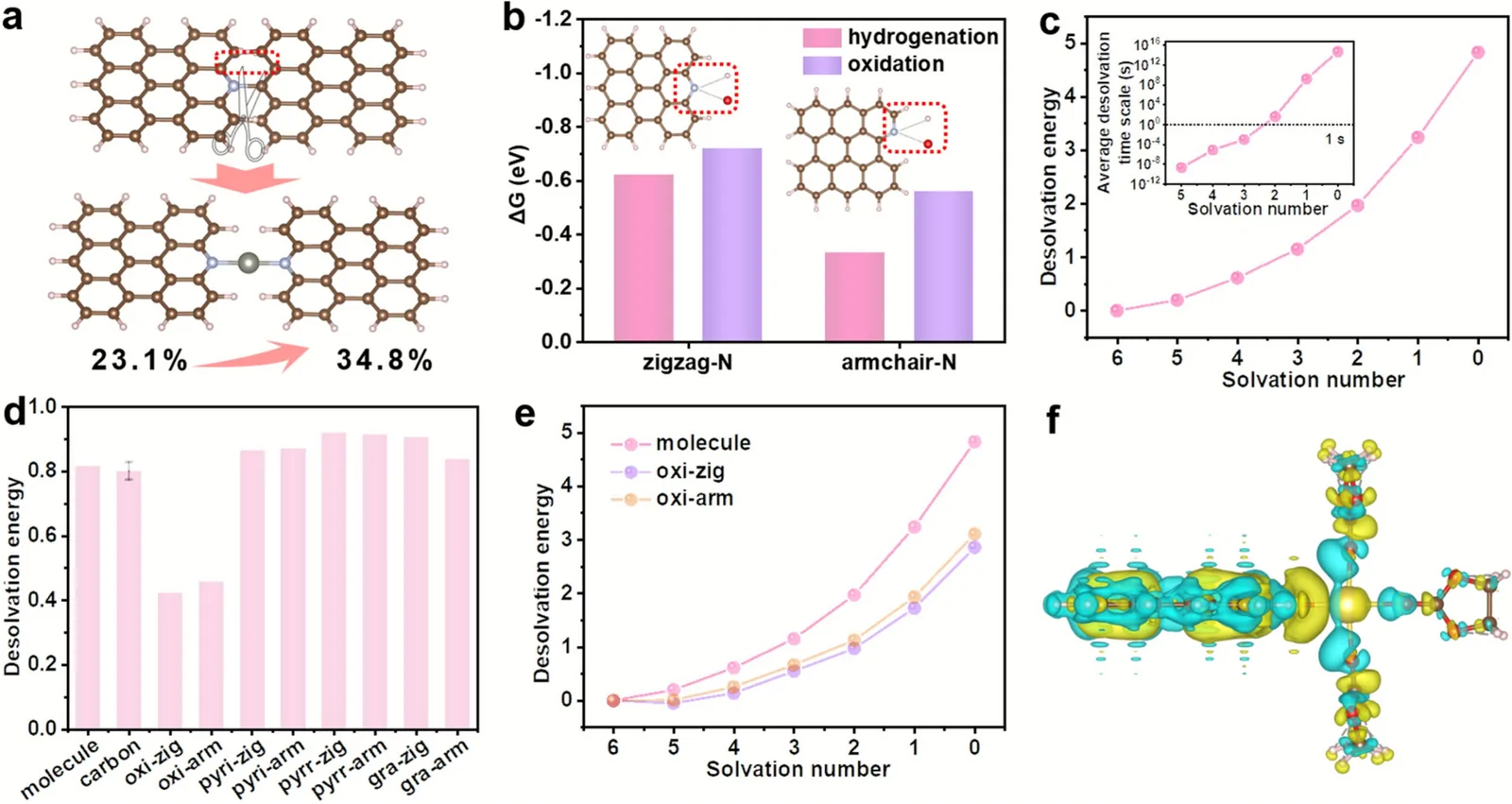
Theoretical simulations on the sodium storage enhancement mechanism. **a** Crystalline structure of N-doped carbon with and without Zn^2+^ as a functional group. **b** Free energy of hydrogenation and oxidation processes in oxidized N with zigzag-N or armchair-N configurations. **c** Desolvation energy of Na^+^ coordinated with EC solvent molecules. **d** Desolvation energy comparison of Na^+^ coordinated with three EC solvent molecules on different N-doped carbons. **e** Desolvation energy comparison of Na^+^ coordinated with EC solvent molecules on different oxidized N. **f** Charge density difference of Na^+^ coordinated with three solvent molecules on zigzag oxidized N

To further insight into the sodium storage enhancement mechanism of MEC_3_, theoretical calculations were performed** (**Fig. 4). Figure 4a demonstrates a substantial increase in oxidized N concentration from 23.1 to 34.8% upon Zn^2+^ incorporation. This enhancement originates from the preferential oxidation of doped N over hydrogenation in the presence of Zn^2+^, as evidenced by both zigzag- and armchair-type N configurations (Fig. 4b). The calculation result confirms that Zn^2+^ coordination induces directional tailoring effects, thereby promoting N oxidation. Subsequently, considering that the in situ and ex situ characterizations, the desolvation effect of Na^+^ ions coordinated with solvent molecules on carbon structure doped with different types of N was simulated by DFT calculations. Figure S26 shows the different tetrahedral and hexahedral coordination structures of Na^+^ ions with EC, DMC, or their mixed solvents, where the solvation energy of Na^+^ in its hexacoordinate structure with EC is the smallest (Fig. S27). The hexacoordinate structure was selected for simulation calculations due to its demonstrated ability to reduce Na⁺ desolvation energy, thereby enhancing reaction kinetics during charge/discharge processes. Figure 4c displays the desolvation energies of Na^+^ coordinated with different EC solvent molecules, where the desolvation capacities of Na^+^ are gradually decreased with the gradual removal of solvent molecules. Noticeably, the desolvation time taken for the removal of the third EC solvent molecule to the second one begins to exceed 1 s, which is considered as the start of the rate-determining step. Then, the desolvation energy of Na^+^ from three EC solvent molecules to two is compared on carbon structures doped with different N species including pristine carbon, pyridinic N, pyrrolic N, graphitic N, and oxidized N (Figs. 4d and S28), where the carbon structures with oxidized N featuring zigzag and armchair configurations demonstrate lower desolvation energies than others, verifying the superiority of oxidized N for desolvation of Na^+^. Moreover, the carbon structures with zigzag and armchair oxidized N also display lower step-wise desolvation energies compared to pure solvent molecules (Figs. 4e and S29), further verifying the advantages of oxidized N to improve the desolvation kinetics of Na^+^ [64]. Figure 4f displays the charge density difference for a carbon structure with the zigzag oxidized N, where Na^+^ is coordinated with three EC molecules. Charge depletion centers (cyan) and electrons are transferred from carbon structure to Na^+^, while the partial charge accumulation centers (yellow) are dispersed on the oxidized N. In addition, the partial density of states (pDOS) and Bader charge analyses for zigzag/armchair oxidized nitrogen configurations (Fig. S30) demonstrate that the N–O moieties exhibit significant electron-donating capability. The above results indicate oxidized N is the most effective structure to decrease desolvation energy of Na^+^ and enhance sodium storage reaction kinetics. Meanwhile, Zn^2+^ ions act as coordination species, directionally tailoring the microdomain of carbon and therefore enhancing the concentration of oxidized N in MEC_3_. Consequently, the ingenious microdomain configuration, characterized by rich oxidized N and moderate closed pores, significantly promotes the sodium-ion storage performance of carbon, which corresponds to the FTIR results.

### Performance of the MEC_3_-based SDIB

**Fig. 5:**
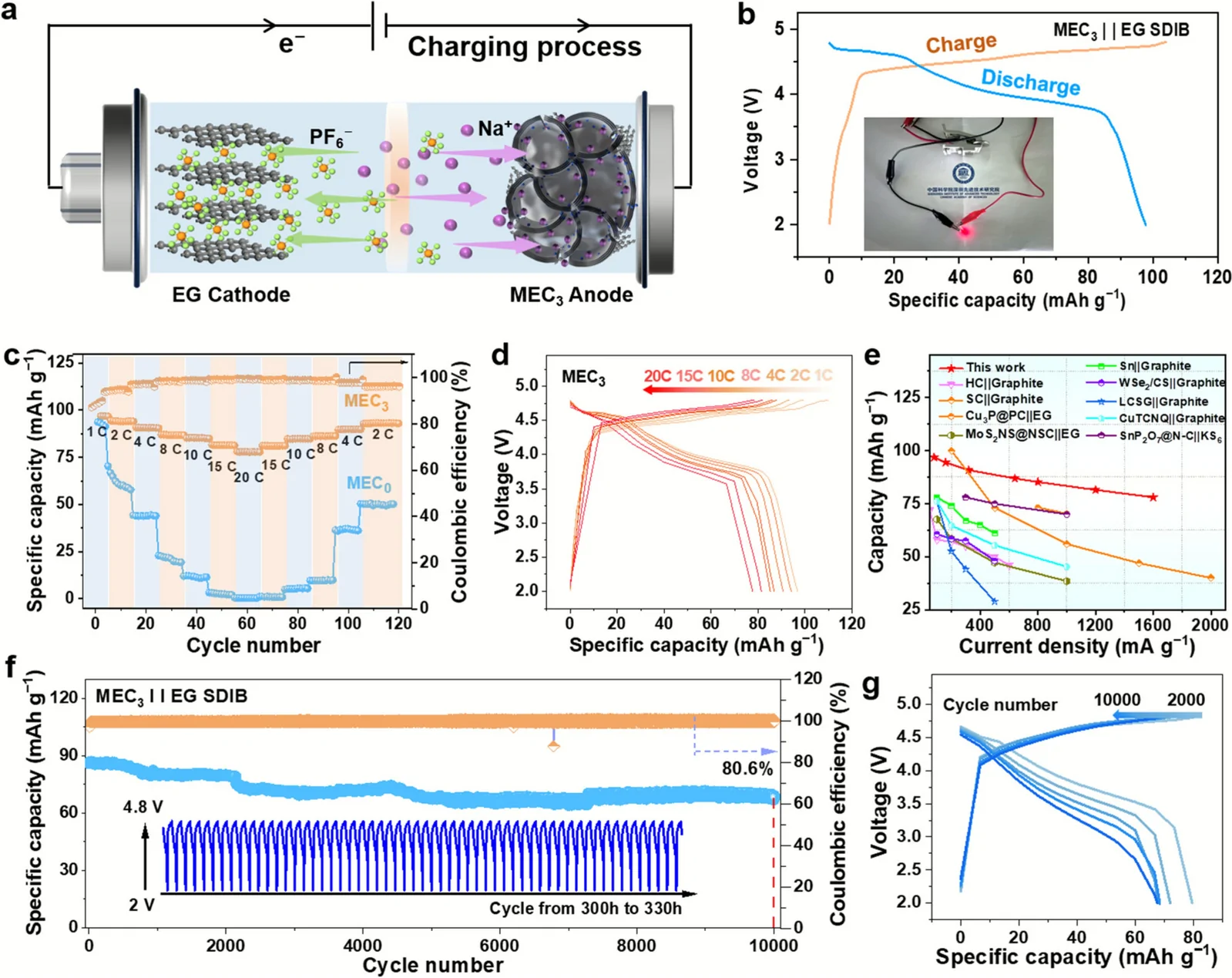
Electrochemical performance of the MEC_3_ || EG SDIB. **a** Schematic illustration of the SDIB configuration. **b** GCD curves of the SDIB at 1 C, with an optical photograph insert lighting up an LED light. **c** Rate capabilities of the MEC_3_ || EG SDIB and MEC_0_ || EG SDIB. **d** GCD curves of MEC_3_ || EG from 1 to 20 C. **e** Comparison of rate performance with previously reported typical SDIBs. **f** Cycling performance of the SDIB at 10 C. **g** GCD curves at 2,000th, 4,000th, 6,000th, 8,000th, and 10,000th cycles

The above results demonstrate that the MEC_3_ exhibits both high plateau capacity and fast Na^+^ ion kinetics in the plateau region, which is beneficial for enhancing the energy density and rate performance of DIB. Therefore, SDIB configuration with MEC_3_ as anode and EG as cathode was further constructed (Fig. 5a). The assembled MEC_3_ || EG SDIB achieves a high capacity of 97 mAh g^− 1^ at 1 C (1 C = 80 mA g^− 1^) with a high working voltage of 4.05 V, which can light up a red LED light once operating (Fig. 5b). As depicted in Fig. 5c, the excellent rate performance of MEC_3_ enables SDIB to maintain capacities of 94, 91, 87, 85, 82, and 78 mAh g^− 1^ at 2, 4, 8, 10, 15, and 20 C, respectively. However, the capacity of MEC_0_-based SDIB significantly decays to 59 mAh g^− 1^ at 4 C, further highlighting the performance advantage of MEC_3_. Particularly, even at an ultra-high rate of 20 C, the MEC_3_-based SDIB still retains a stable charge–discharge voltage plateau and minimal polarization, attributed to the stable sodium storage and rapid sodium storage kinetics in the MEC_3_ plateau region (Fig. 5d). In contrast, the MEC_0_-based SDIB experiences a significant polarization at a rate of above 2 C, rendering normal operation challenging beyond 10 C (Fig. S31). Thanks to the strategic kinetic design of the plateau region, the MEC_3_-based SDIB currently offers the excellent rate performance (Fig. 5e, Table S8) [65–73]. The MEC_3_-based SDIB effectively balances the trade-off between energy density and power density, delivering a maximum energy density of 222 Wh kg^− 1^ with a corresponding power density of 383 W kg^− 1^. Remarkably, even when the power density increases by nearly an order of magnitude to 3530 W kg^− 1^, the energy density remains as high as 177 Wh kg^− 1^. These values compare favorably with recently reported sodium-ion and dual-ion batteries (Table S9) [30, 32, 72, 74–76], where high-energy density is often achieved only at low-power density or vice versa. Furthermore, the MEC_3_-based SDIB also demonstrates superior cycle stability, with capacity retention of 80.6% after 10,000 cycles at 10 C (Fig. 5f) with robust charge/discharge curves (Fig. 5g) and stable medium voltage with an average value of 3.68 V (Fig. S32), which is the wonderful performance among the reported SDIBs. Although slight capacity fluctuations are observed likely arising from detachment, contact loss of active materials [77], or temperature variation in the laboratory, the battery still exhibits outstanding long-term cycling stability under high-rate operation.

## Conclusion

In conclusion, this work presents an innovative carbon microdomain engineering strategy, which entails molecular-scale regulation of closed-pore structures and the construction of high-activity defects to enhance the sodium-ion storage capacity and kinetics of carbon materials. Specifically, acetate serving as a pore-forming agent and Zn^2+^ ions as coordination species induce the formation of high-activity N species within micrographene domains and generate unique closed pores. Electrochemical characterizations and theoretical calculations confirm that the synergetic design of closed-pore structures and high-active N species effectively accelerates the sodium-ion desolvation, therefore promoting sodium storage kinetics. Correspondingly, the optimized carbon material MEC_3_ significantly enhances plateau-region kinetics and mitigates the deterioration of plateau capacity at high rates. The MEC_3_ anode delivers a reversible capacity of up to 427 mAh g^− 1^ at 0.1 C and a high plateau capacity of 253 mAh g^− 1^ even at 1 C, which ranks among the top performances of carbonaceous anodes reported to date. Moreover, benefiting from the advantages of MEC_3_, the assembled SDIB exhibits outstanding ultralong-term cycling stability and superior rate capability with high capacity retention of 80.6% after 10,000 cycles at 10 C, which represents best performance among the SDIB systems. This strategy paves a new way for the design of high-performance carbonaceous materials and provides a novel perspective for the development of dual-ion battery systems.

## Supplementary Information

Below is the link to the electronic supplementary material.

Supplementary file 1 (DOCX 5356 KB)
