# Supplementary material for: Ion-Mediated Carbon Microdomain Engineering Boosting Enhanced Plateau Capacity of Carbon Anode under High Rate Towards High-Performance Sodium Dual-Ion Batteries
Source: Nanomicro Lett. 2026 Jan 5;18:161. doi: 10.1007/s40820-025-02008-4 (PMC12765788; doi:10.1007/s40820-025-02008-4)
Supplement: Supplementary file 1 — (DOCX 5356 KB) [file 40820_2025_2008_MOESM1_ESM.docx]

Supporting Information for

**Ion-Mediated Carbon Microdomain Engineering Boosting Enhanced Plateau Capacity of Carbon Anode under High Rate Towards High-Performance Sodium Dual-Ion Batteries**

Bin Tang^1,2^^#^, Yuchen Zhang^1,2#^, Bifa Ji^1#^, Geng Yu^1,4^, Yongping Zheng^1^, Xiaolong Zhou^1,2^, Nuntaporn Kamonsutthipaijit^3^, Pornsuwan Buangam^3^, Sarayut Tunmee^3^, Hideki Nakajima^3^, Ukit Rittihong^3^, Qingguang Pan^1,2^*, Fan Zhang^1,2^*, and Yongbing Tang^1,2^*

^1^ Advanced Energy Storage Technology Research Center, Shenzhen Institutes of Advanced Technology, Chinese Academy of Sciences, Shenzhen 518055, P. R. China

^2^ University of Chinese Academy of Sciences, Beijing 100049, P. R. China

^3^ Synchrotron Light Research Institute (Public Organization), 111 University Avenue, Muang District, Nakhon Ratchasima 30000, Thailand

^4^ Southern University of Science and Technology, Shenzhen 518055, P. R. China

^#^Bin Tang, Yuchen Zhang, and Bifa Ji contributed equally to this work.

*Corresponding authors. E-mail: [fan.zhang1@siat.ac.cn](mailto:fan.zhang1@siat.ac.cn) (Fan Zhang); [qg.pan@siat.ac.cn](mailto:qg.pan@siat.ac.cn) (Qingguang Pan); [tangyb@siat.ac.cn](mailto:tangyb@siat.ac.cn) (Qingguang Pan)

**S1 Fitting and analysis of SAXS**

The background was deducted by the SAXSIT code developed by the Synchrotron light research institute - SLRI BL1.3L to obtain the 1D scattering profile of the sample as function of azimuthal angle. The pore structure information was obtained by fitting the scattering intensity (Q)-scattering intensity curve. Modified porous equation is given as [S1]:

I(q)=$I\left( q \right)=\frac{A}{q^{a}}+\frac{B^{'}a_{1}^{6}\Delta\rho^{2}}{{(1+a_{1}^{2}Q^{2})}^{2}}+\frac{C}{{(1+a_{2}^{2}Q^{2})}^{2}}+D$ （S1）

Here, A and B are the pore specific surface area parameters; A and B are proportional to the total surface area of the large and small pores, respectively. a is the slope of the initial drop of the intensity in the logarithmic plot. $a_{1}$ is characteristic length over which the scattering power changes. Based on the assumption that the pore structure was spherical with the radius of a spherical pore volume by $R=a_{1}\sqrt{10}$. Δρ is the electron density contrast, sensitive to changes in the electron density of the pores. $a_{2}$ is characteristic length of an additional intermediate pore size. C is proportional to the surface area of the pores with the intermediate size. D is a constant background term.

Assuming that the pore structure was spherical, the average pore size $a_{1}\sqrt{10}$ and the specific surface area parameter B of the small pores were calculated by the equation S1 in the case of a small number of open pores. The closed-pore volume parameter ($V$) was defined, which can be calculated by S2~S3:

$k\cdot B=4\pi\left( a_{1}\sqrt{10} \right)^{2}\cdot x$ (S2)

$V=\frac{4}{3}\pi\cdot\left( a_{1}\sqrt{10} \right)^{3}\cdot x=\frac{\sqrt{10}}{3}a_{1}\cdot B$ (S3)

Here, $k$ is the specific surface area factor of the pores, and the specific surface area of the pores is $k\cdot B$. $x$ represents the number of pores.

**S2 Electrochemical kinetic analysis**

Cyclic voltammetry (CV) cures were used to measure the electrochemical reaction and kinetics of MECs in half-cell structure at different scan rates of 0.1, 0.3, 0.5, 1.0, 3.0, and 5.0 mV s^−1^. According to the power law, the current response at a fixed potential has a linear or exponential relationship with the applied scan rate [S2]:

$i=a\cdot v^{b}$ (S4)

Here, $v$ (mV s^−1^) is the scan rate, $i$ (mA) is the response current measured at a fixed potential using a specific scan rate, a and b are variable parameters.

**S3 The energy density and power density**

The energy density and power density of MEC_3_ || EG were calculated on the basis of the active materials of both electrodes. The medium discharge voltage ($\bar{V}$) at different current densities was obtained from the galvanostatic charge–discharge profiles. The MEC_3_ anode had a diameter of 12 mm (corresponding to an area *S*_1_=0.36π cm^2^) with a mass loading of approximately 1 mg cm^−2^, while the EG cathode had a diameter of 10 mm (corresponding to an area *S*_2_=0.25π cm^2^) with a mass loading of approximately 2 mg cm^−2^. Accordingly, the gravimetric energy density (ED) and power density (PD) normalized to the total active mass of both electrodes were calculated according to Equations (S5) and (S6).

$E=\frac{C\cdot\bar{V}\cdot S_{2}\cdot L_{2}}{S_{1}\cdot L_{1}+S_{2}\cdot L_{2}}$ （S5）

$P=\frac{C\cdot\bar{V}\cdot S_{2}\cdot L_{2}}{\left( S_{1}\cdot L_{1}+S_{2}\cdot L_{2} \right)\cdot t}$ （S6）

Herein, *C* and *t* denote the specific capacity of the cathode at a given current density and the corresponding discharge time, respectively. *L*_1_ and *L*_2_ are corresponding to the areal mass loadings of the active materials in the MEC_3_ anode and the EG cathode, respectively. Based on these parameters, the energy density and power density of MEC_0_ and MEC_3_ were calculated under various current densities, and the results were systematically compared with those reported in recent literature.

**Supplementary Tables**

**Table S1** Comparison of different carbon microstructure regulation strategies in terms of structural features, capacity and rate performance

| Ref.MS/SI | Structural Regulation Strategy | Performance |
| --- | --- | --- |
| [30]/ [S3] | The free radical–induced strategy by controlled delignification regulating radical release to promote closed pore formation in hard carbon | Capacity of 350 mAh g⁻^1^@20 mA g⁻^1^; plateau capacity of 241 mAh g⁻^1^@20 mA g⁻^1^ |
| [31]/ [S4] | Converting open pores into closed ones via waste mask–derived carbon deposition for enhanced sodium storage | Capacity of 335.5 mAh g⁻^1^@20 mA g⁻^1^; plateau capacity of 180 mAh g⁻^1^@300 mA g⁻^1^ |
| [32]/ [S5] | Activated carbon micropores filled with graphitic-like domains via self-catalyzed chemical vapor deposition | Capacity of 435.5 mAh g⁻^1^@20 mA g⁻^1^; plateau capacity of 200 mAh g⁻^1^@300 mA g⁻^1^ |
| [33]/ [S6] | Steric hindrance in polymer precursor to promote closed-pore formation | Capacity of 340.3 mAh g⁻^1^@ 30 mA g⁻^1^; plateau capacity of 200 mAh g⁻^1^@300 mA g⁻^1^ |
| [37]/ [S7] | One-step Zn-gluconate pyrolysis to form closed pores by ZnO etching carbon materials | Capacity of 481.5 mAh g⁻^1^@20 mA g⁻^1^; plateau capacity of 180 mAh g⁻^1^@200 mA g⁻^1^ |
| [15]/ [S8] | Pre-desolvation by extending the aging time to generate a highly aggregated electrolyte configuration inside the nanopore | Capacity of 319.7 mAh g⁻^1^@20 mA g⁻^1^; plateau capacity of 250 mAh g⁻^1^@100 mA g⁻^1^ |
| [34]/ [S9] | The structural regulation strategy combines self-templating, high-temperature reconstruction, and soft-carbon coating to tailor pore architecture and optimize graphene nanodomains in hard carbon. | Capacity of 414 mAh g⁻^1^@20 mA g⁻^1^; plateau capacity of 200 mAh g⁻^1^@200 mA g⁻^1^ |
| [36]/ [S10] | Zinc-assisted cross-linking and etching precisely regulate epoxy-resin-derived hard carbon to form semi-closed ultramicropores for rapid Na⁺ storage | Capacity of 408 mAh g⁻^1^@30 mA g⁻^1^; plateau capacity of 256.2 mAh g⁻^1^@30 mA g⁻^1^ |
| This work | Ion-mediated microdomain engineering to build molecular-scale closed pores and enrich oxidized-N sites | Capacity of 427 mAh g⁻^1^@30 mA g⁻^1^; high plateau capacity of 253 mAh g⁻^1^@300 mA g⁻^1^ |

**Table S2** The specific surface area and pore diameter distribution for MEC_0_~MEC_4_

| Samples | BET Surface Area  (m^2^ g^−1^) | t-Plot micropore volume  (cm^3^ g^−1^) |
| --- | --- | --- |
| MEC_0_ | 8.69 | 0.002 |
| MEC_1_ | 13.26 | 0.005 |
| MEC_2_ | 18.95 | 0.004 |
| MEC_3_ | 39.13 | 0.009 |
| MEC_4_ | 229.73 | 0.071 |

**Table S3** The fitted FWHM of (002) and (100) peaks in XRD patterns

| Samples / FWHM | peak (002) | peak (100) |
| --- | --- | --- |
| MEC_0_ | 7.090 | 4.104 |
| MEC_1_ | 6.420 | 4.010 |
| MEC_2_ | 6.300 | 3.928 |
| MEC_3_ | 6.108 | 3.001 |
| MEC_4_ | 6.850 | 3.958 |

**Table S4** Each parameter after SAXS fitting

| Samples / parameter | A | a | B | $a_{1}$ | V |
| --- | --- | --- | --- | --- | --- |
| MEC_0_ | 13.391 | 4.196 | 22860.693 | 0.339 | 8161.327 |
| MEC_1_ | 14.789 | 4.008 | 48199.254 | 0.316 | 16077.822 |
| MEC_2_ | 89.930 | 3.778 | 76643.527 | 0.327 | 26397.306 |
| MEC_3_ | 122.572 | 3.313 | 79320.162 | 0.411 | 34395.214 |
| MEC_4_ | 206.430 | 2.926 | 23488.288 | 0.442 | 10953.631 |
| (MEC_3_-Discharge-0.0001V) | 102.405 | 3.881 | 21545.206 | 0.392 | 8902.571 |

**Table S5** ICP results of element contents of Zn and N in MEC_0_ and MEC_3_

| Samples | N (wt%) | Zn (wt%) |
| --- | --- | --- |
| MEC_0_ | 0.97 | 0 |
| MEC_3_ | 1.154 | 0.00155 |

**Table S6** Comparison of the key testing parameters of sodium-half cell (Corresponding to Fig. 2e)

| Half-cell  Na \|\| carbon materials | Electrolyte | Loading  （mg cm^-2^） | Charge–discharge voltage range（V) | Ref.MS/SI |
| --- | --- | --- | --- | --- |
| MEC_3_ | 1 M NaClO_4_ in EC/DMC (1:1 v/v) | 1 – 1.5 | 0.0001 – 2.0 | This work |
| HG1300 | 1 M NaClO_4_ in EC/DMC (1:1 v/v) | 1.5 – 2.0 | 0.001 – 2.5 | [46]/ [S2] |
| HC | 1 M NaClO_4_ in EC/DEC (1:1 v/v) with 2 vol% FEC | 0.8 – 1.1 | 0.002 – 3.0 | [37]/ [S7] |
| HC | 1 M NaPF_6_ in EC/DEC/EMC (1:1:1 v/v/v) with 5 vol% FEC | ~1.0 | 0.01 – 3.0 | [15]/ [S8] |
| 4S-HCNs | 1 M NaClO_4_ in EC/DEC (1:1 v/v) with 5 vol% FEC | / | 0.001 – 2.0 | [47]/ [S11] |
| CPs | 1 M NaClO₄ in PC with 5 vol% FEC | / | 0.01 – 3.0 | [48]/ [S12] |
| FP-MP | 1 M NaClO_4_ in EC/DMC (1:1 v/v) | 1.5 – 2.0 | 0 – 2.5 | [49]/ [S13] |
| HC-1300 | 1 M NaClO_4_ in EC/DEC (1:1 v/v) with 5 vol% FEC | ~2.5 | 0.001 – 2.0 | [50]/ [S14] |
| HC | 1 M NaClO_4_ in EC/PC (1:1 v/v) | 1.0 – 2.3 | 0.02 – 3.0 | [51]/ [S15] |
| HCS | 1 M NaPF_6_ in EC/DEC (1:1 v/v) | ~2.0 | 0.01 – 1.5 | [52]/ [S16] |

**Table S7** The physical parameters of each component in the EIS equivalent circuit diagram of MEC_0_ and MEC_3_ at 25 °C

| Sample | MEC_0_ (Ω) | MEC_3_ (Ω) |
| --- | --- | --- |
| *R*_s_ | 2.67 | 2.11 |
| *R*_ct1_ | 56.16 | 29.45 |
| *R*_ct2_ | 70.44 | 14.66 |

**Table S8** Comparison of the key testing parameters of SDIBs (Corresponding to Fig. 5e)

| Cell configuration  Anode \|\| Cathode | Electrolyte | Loading  （mg cm^-2^） | Charge–discharge voltage range（V) | Ref.MS/SI |
| --- | --- | --- | --- | --- |
| HC \|\| EG | 1 M NaPF_6_ in EC/DMC/EMC (1:1:1) | 2 | 2.0 – 4.8 | This work |
| HC \|\| Graphite | 0.8 M NaPF_6_ in PC | / | 1.5 – 4.8 | [65]/ [S17] |
| SC \|\| Graphite | 1 M NaPF_6_ in EC/DEC | / | / | [66]/ [S18] |
| Cu_3_P@PC \|\| EG | 1 M NaPF_6_ in EC/EMC/DMC (1:1:1) | 0.5 | 2.0 – 5.0 | [67]/ [S19] |
| MoS_2_NS@N \|\| EG | 1 M NaPF_6_ in EC/EMC/DMC (1:1:1) | / | 1.0 – 4.0 | [68]/ [S20] |
| Sn \|\| Graphite | / | / | 2.0 – 4.8 | [73]/ [S21] |
| WSe₂/CS \|\| Graphite | 1 M NaPF_6_ in EC/EMC/DMC (1:1:1) | 1.0 – 4.5 | 0 – 4.0 | [69]/ [S22] |
| LCSG \|\| Graphite | 1 M NaPF_6_ in EC/EMC (1:1) | / | 2.0 – 5.0 | [70]/ [S23] |
| CuTCNQ \|\| Graphite | 2 M NaPF_6_ in EC/DMC (1:1) | 0.6 | 2.0 – 4.8 | [71]/ [S24] |
| SnP_2_O_7_@N \|\| KS_6_ | 1 M NaPF_6_ in EC/DMC/EMC (4:3:2) | / | 1.0 – 4.0 | [72]/ [S25] |

**Table S9** Comparison of energy density and the corresponding power density of different sodium storage systems (based on the active materials of both electrodes)

| Cell configuration  Anode \|\| Cathode | Energy density  (Wh kg⁻^1^) | Power density  (W kg⁻^1^) | Medium  Voltage (V) | Ref.MS/SI |
| --- | --- | --- | --- | --- |
| HC \|\| EG | 222 | 383 | 4.05 | This work |
|  | 203 | 1560 | 4.03 |  |
|  | 200 | 1997 | 4.01 |  |
|  | 188 | 2804 | 3.96 |  |
|  | 177 | 3530 | 3.89 |  |
| HC \|\| Na_3_V_2_(PO_4_)_3_ | 209 | 19 | ~3.35 | [75]/ [S26] |
|  | 182 | 2172 | / |  |
| HC \|\| Na_3_V_2_(PO_4_)_3_ | 157 | 3800 | ~3.10 | [74]/ [S27] |
| HC \|\| Na_3_V_2_(PO_4_)_3_ | 181 | / | / | [76]/ [S28] |
| HC \|\| Na_3_V_2_(PO_4_)_3_ | 266 | / | ~3.25 | [62]/ [S29] |
| HC \|\| Na_3_V_2_(PO_4_)_3_ | 278 | / | ~3.20 | [31]/ [S4] |

**Supplementary Figures**

**
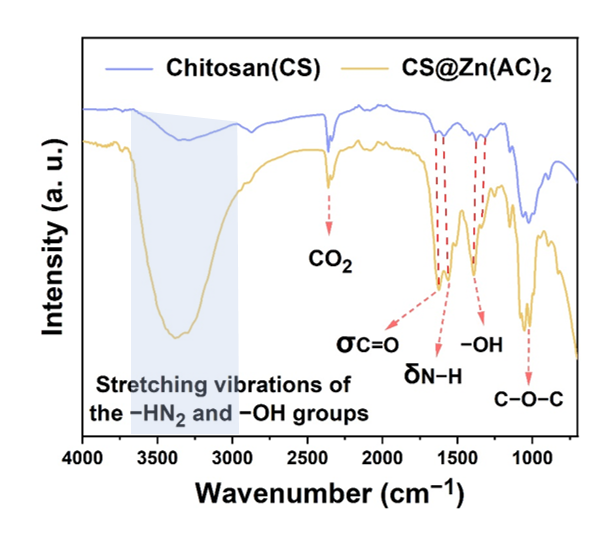
**

**Fig. S1** FTIR Spectra of chitosan and CS@Zn(AC)_2_

**
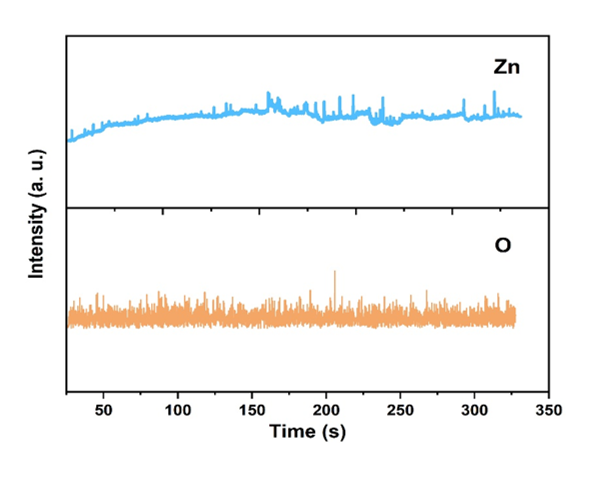
**

**Fig. S2** GDOES spectra of zinc and oxygen in CS@Zn(AC)_2_


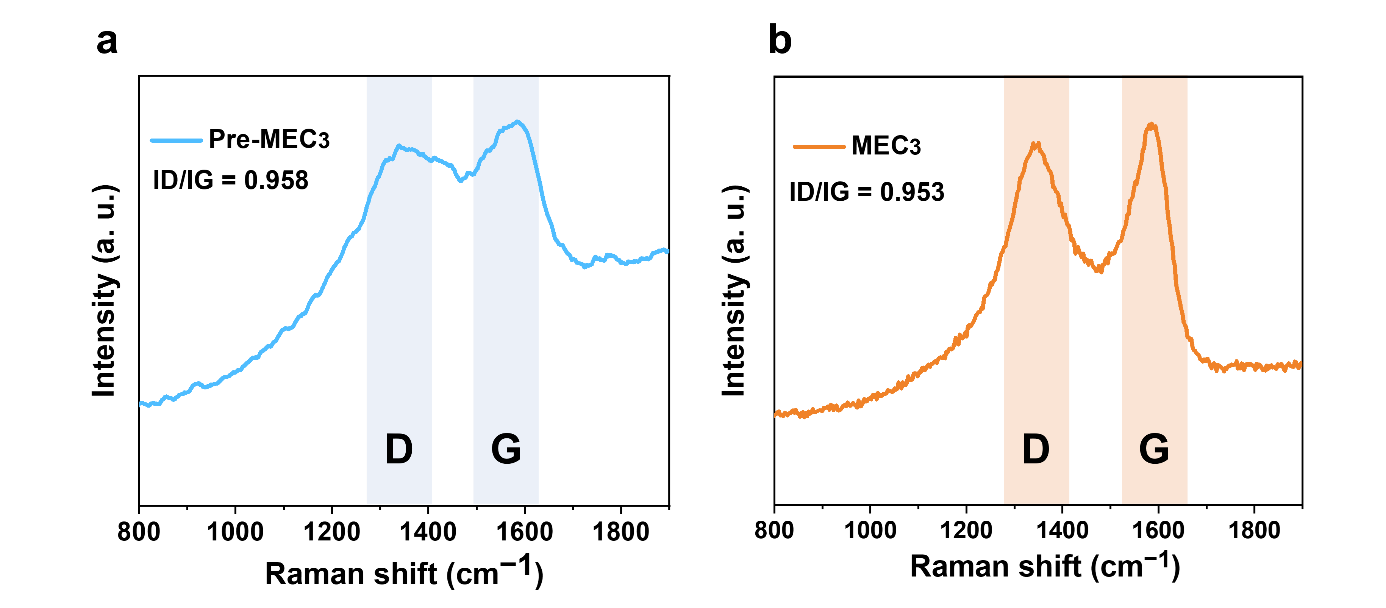


**Fig. S3** Raman spectra of the CS@Zn(AC)_2_ pre-carbonized product and MEC_3_


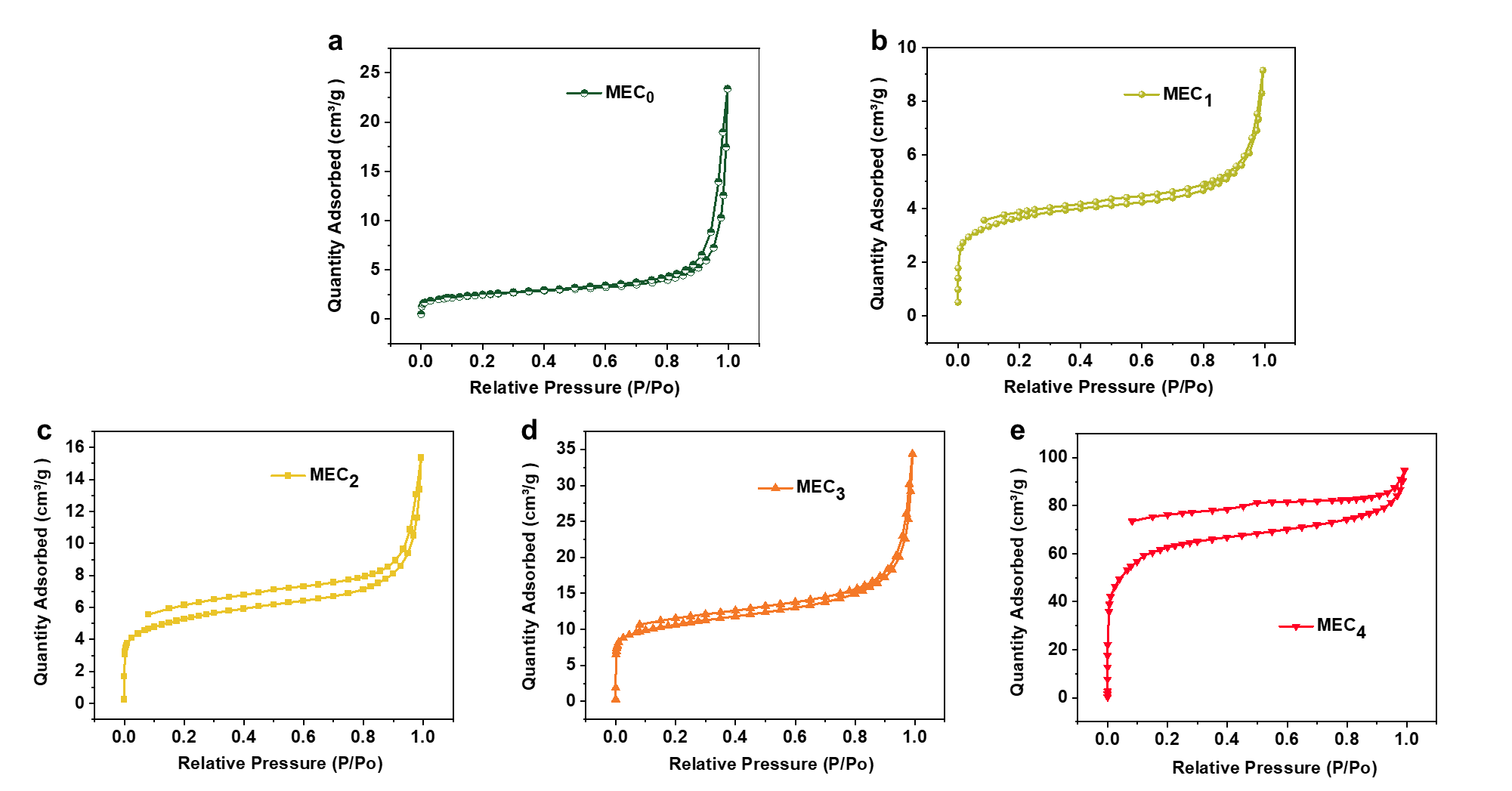


**Fig. S4** BET data of MEC_0_ ~ MEC_4_


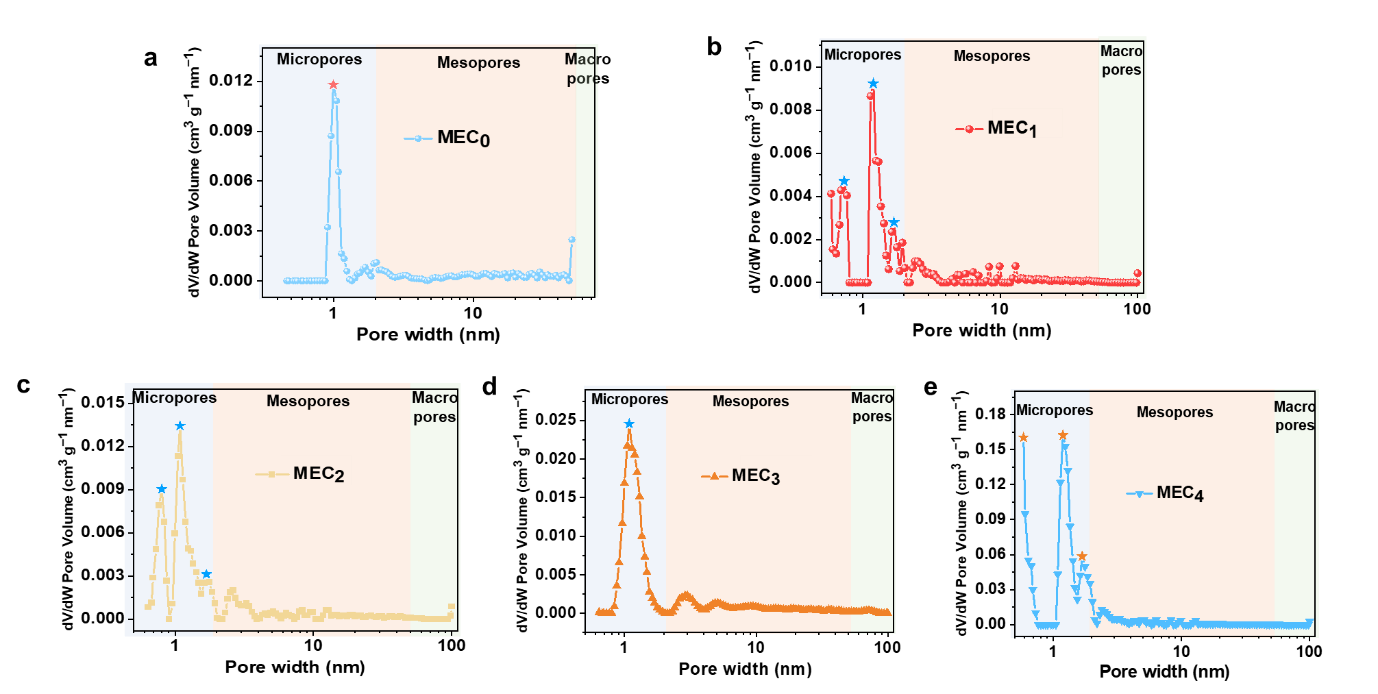


**Fig. S5** The pore diameter distribution curves of MEC_0_~ MEC_4_

**
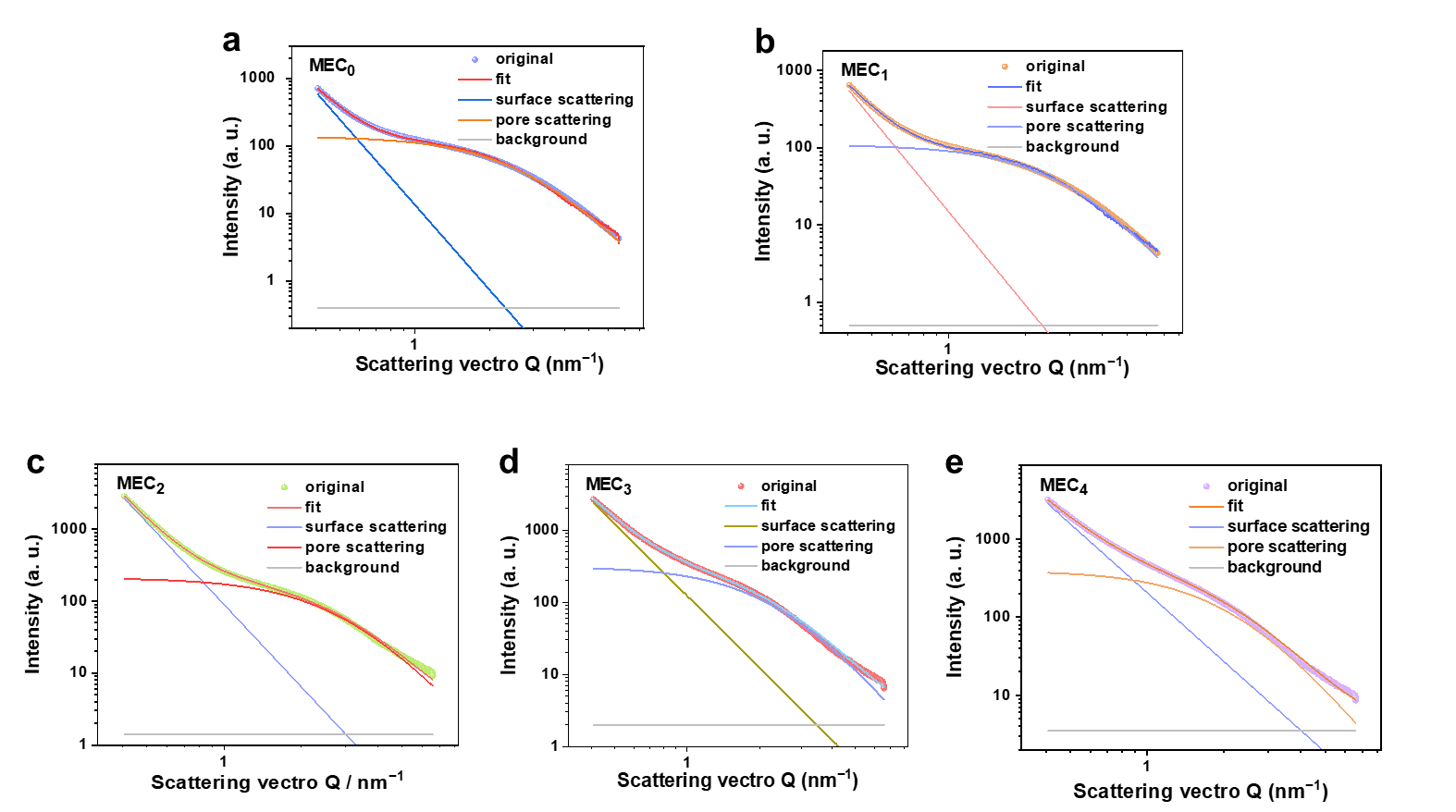
**

**Fig. S6** The fitted SAXS patterns of MEC_0_~ MEC_4_


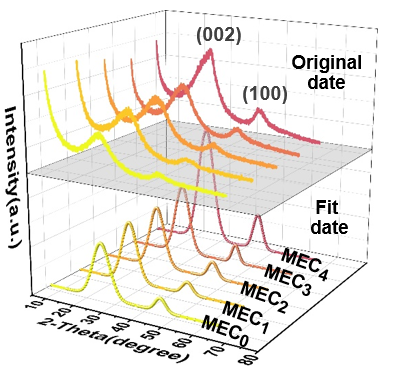


**Fig. S7** XRD patterns for MEC_0_ ~ MEC_4_

**
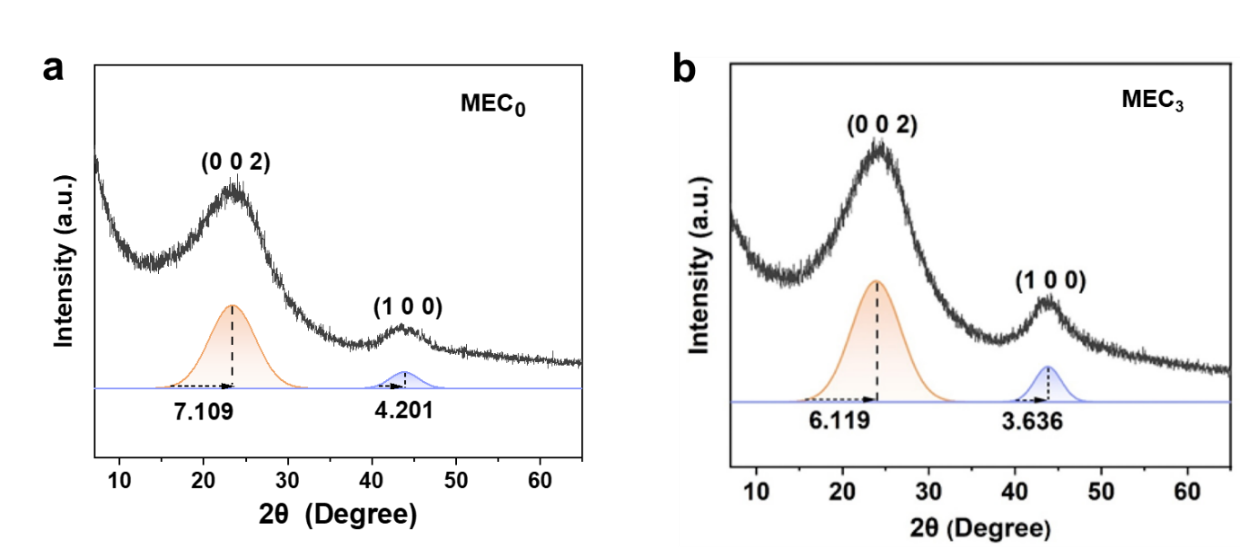
**

**Fig. S8** The fitted XRD patterns of MEC_0_ and MEC_3_


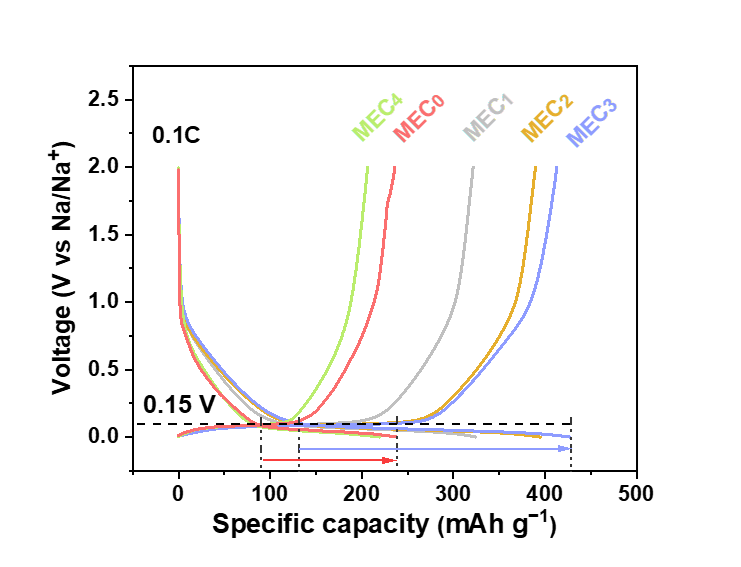


**Fig. S9** The galvanostatic charge and discharge curves for MEC_0_ ~ MEC_4_ at 0.1 C


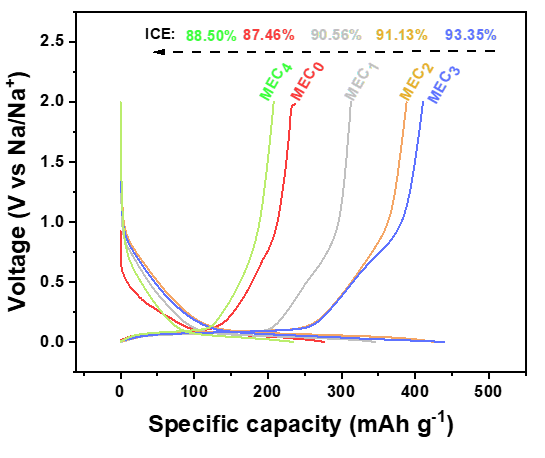


**Fig. S10** The galvanostatic charge and discharge curves and initial Coulombic efficiency during the first cycle for MEC_0_ ~ MEC_4_ at 0.1 C


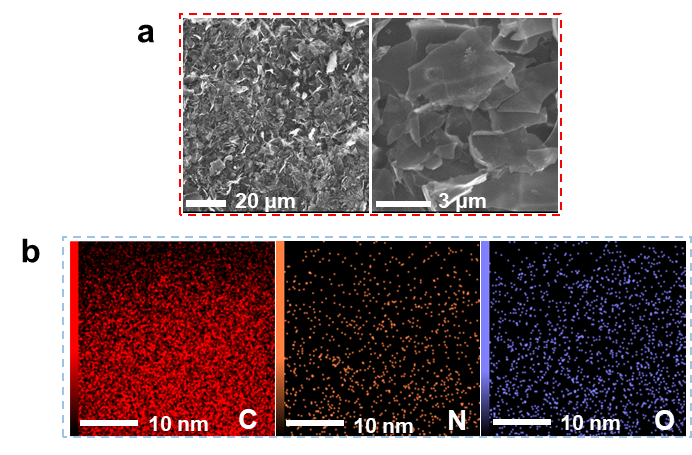


**Fig. S11** **a** SEM images of MEC_3_ and **b** corresponding EDS mapping images





**Fig. S12** XPS spectrum of Zn 2p for MEC_3_

**
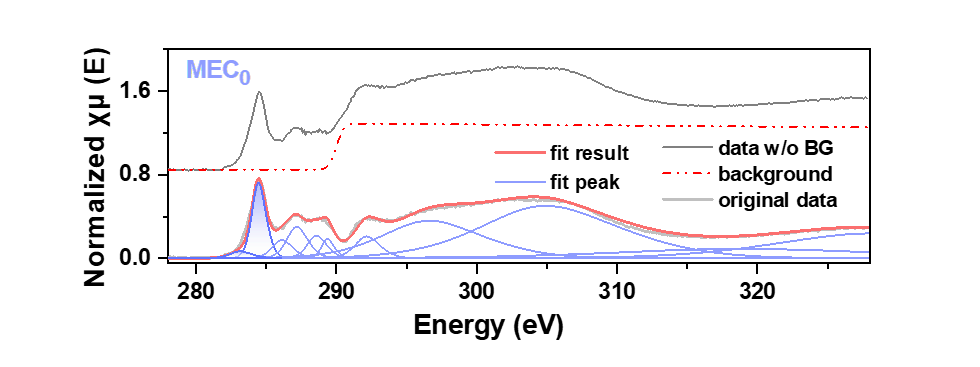
**

**Fig. S13** The carbon K-edge NEXAFS spectra of MEC_0_ in the energy range of 275~335 eV


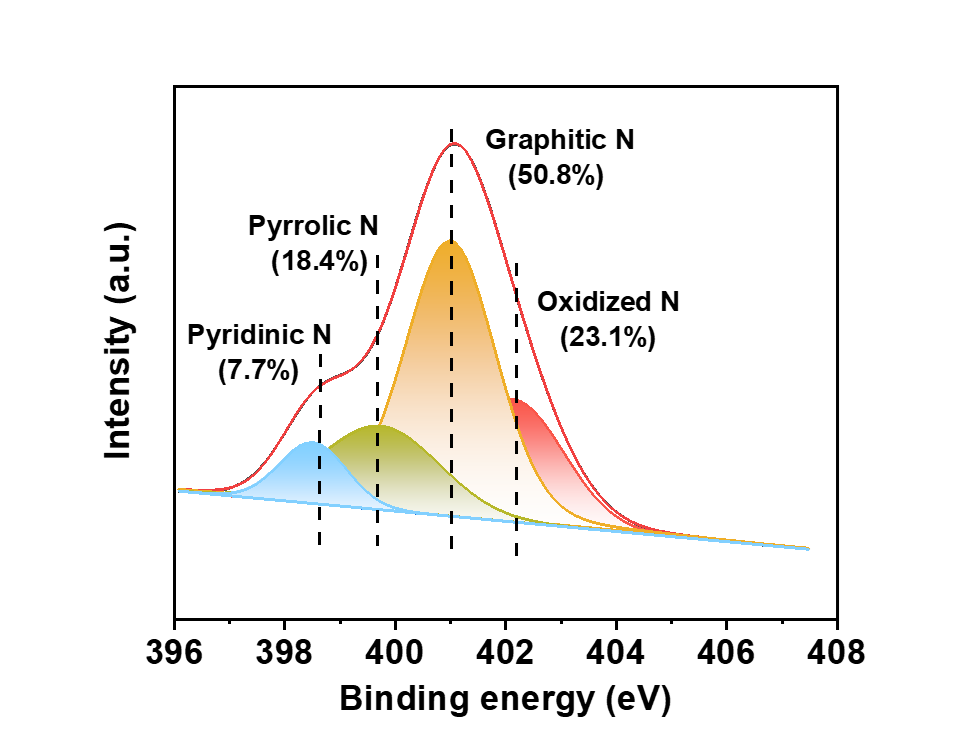


**Fig. S14** XPS spectra of N 1s for MEC_0_


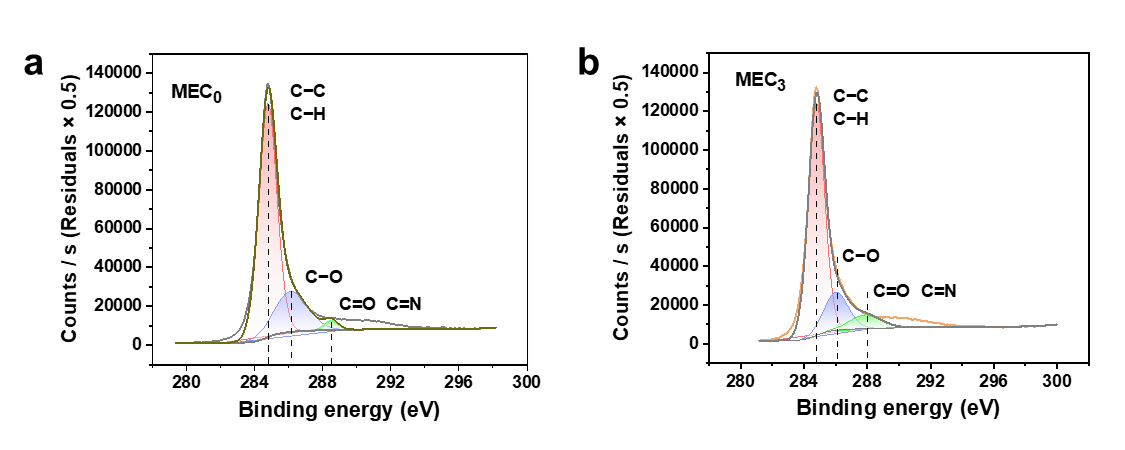


**Fig. S15** XPS spectra of C 1s for **a** MEC_0_ and **b** MEC_3_


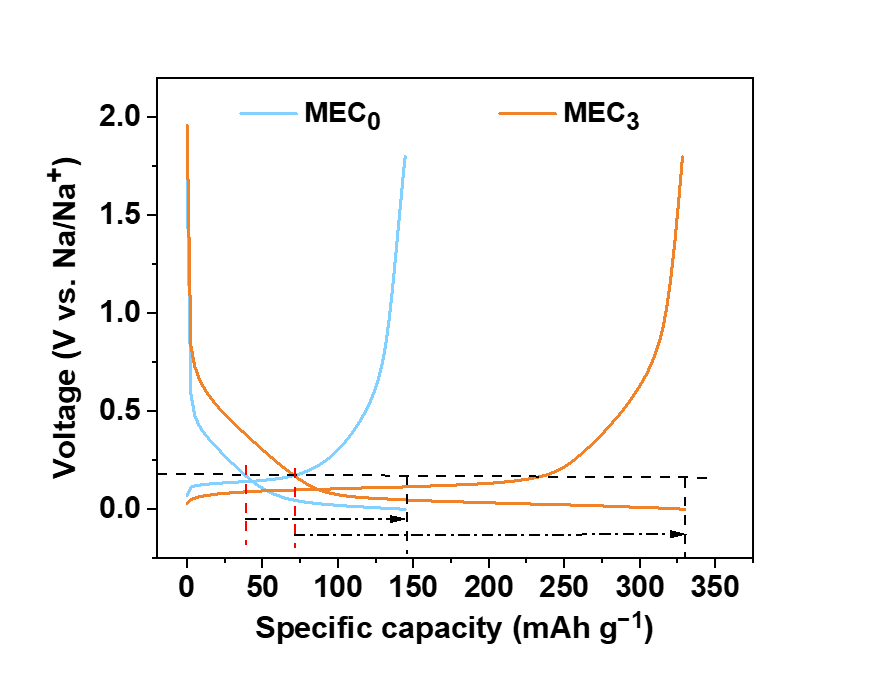


**Fig. S16** Comparison of GCD curves and plateau capacity of MEC_0_ and MEC_3_ at 1 C


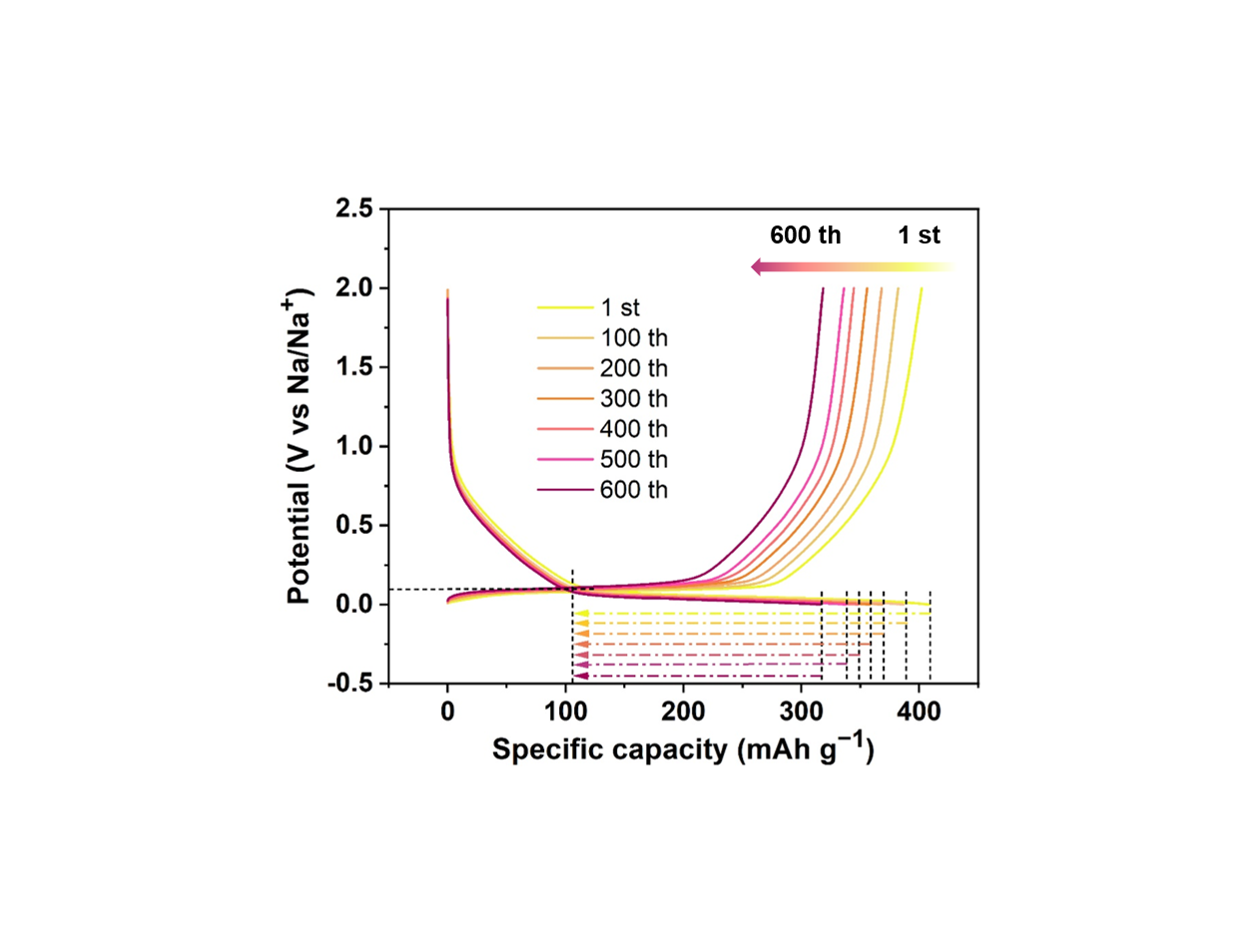


**Fig. S17** The GCD curves of MEC_3_ at 0.2 C under 1~600 cycles. Before the 0.2 C long-term cycling, the half-cell underwent activation through 2 cycles at 0.1 C


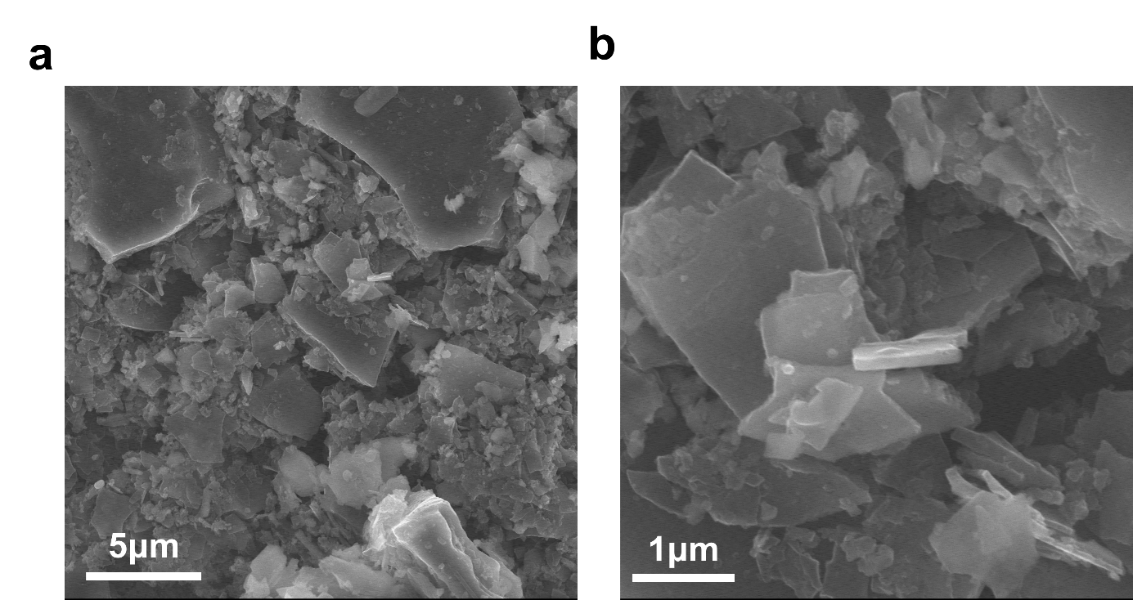


**Fig. S18** SEM images of MEC_3_ after 100 cycles

**
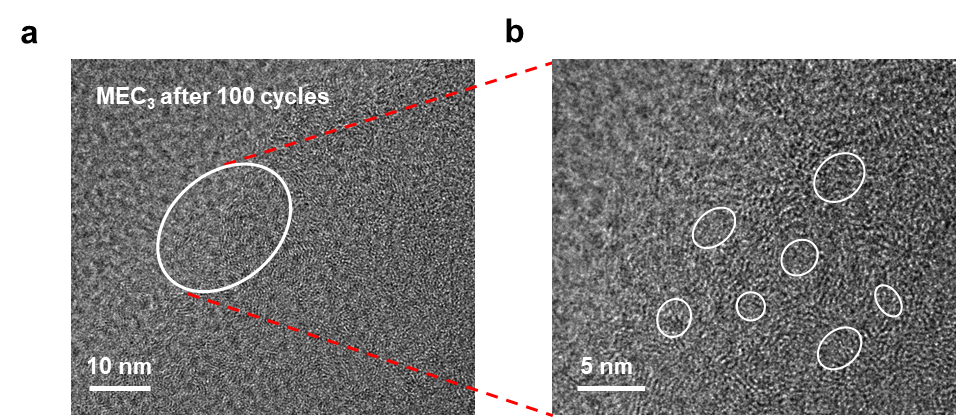
**

**Fig. S19** HR-TEM images of MEC_3_ after 100 cycles

**
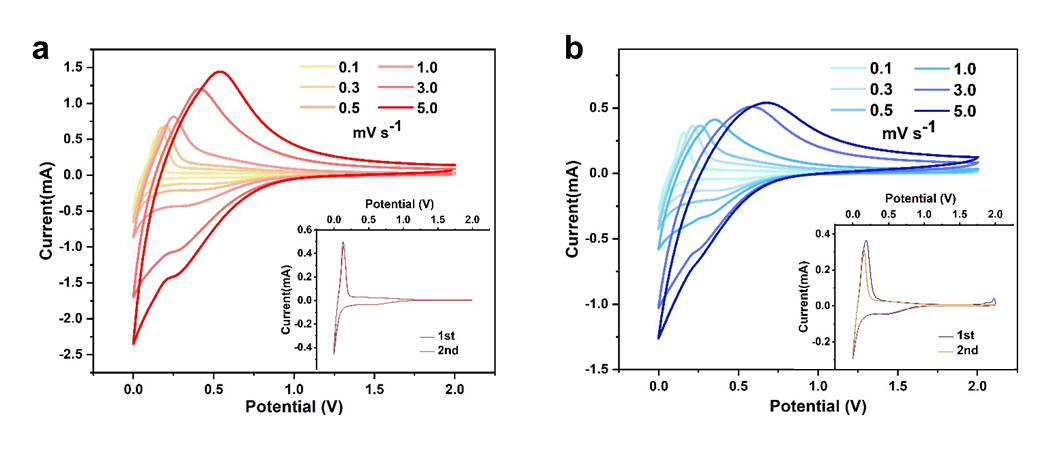
**

**Fig. S20** CV curves of **a** MEC_3_ and **b** MEC_0_ at scan rates of 0.1~5 mV s^−1^


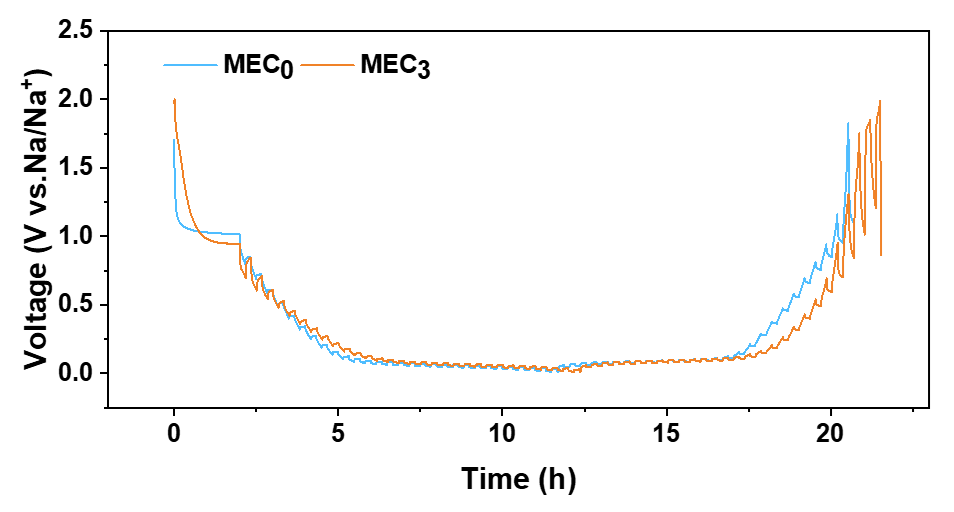


**Fig. S21** The GITT potential profiles of MEC_3_ and MEC_0_

**
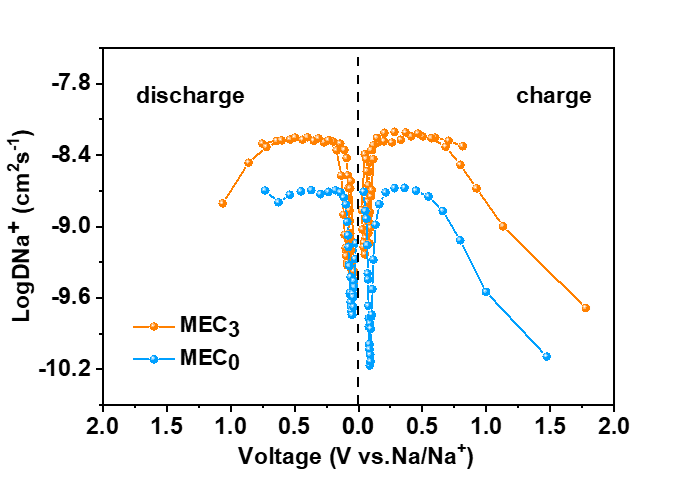
**

**Fig. S22** Na⁺ diffusion coefficients of MEC_3_ and MEC_0_ derived from GITT measurements


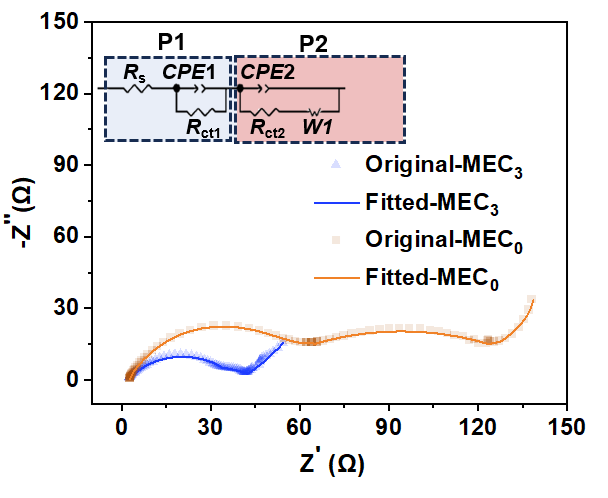


**Fig. S23** Nyquist curves and equivalent circuit diagrams of MEC_3_ and MEC_0_ at 25 °C

**
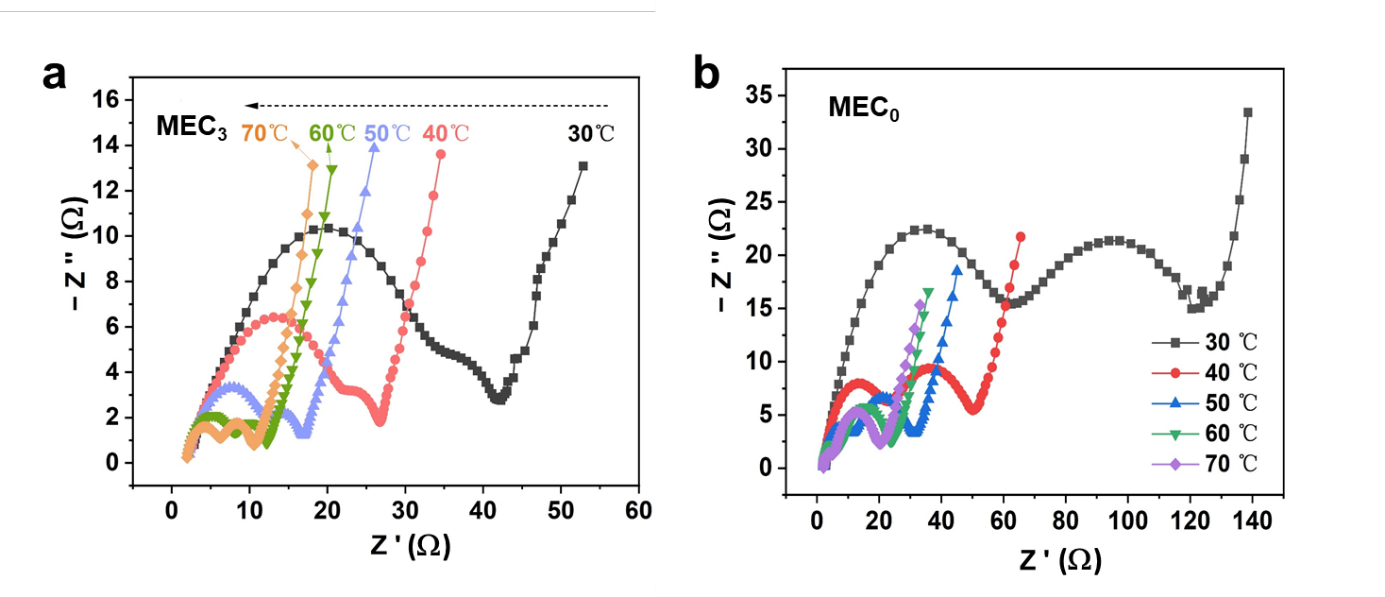
**

**Fig. S24** Nyquist curves and equivalent circuit diagrams of **a** MEC_3_ and **b** MEC_0_ at 30 °C, 40 °C, 50 °C, 60 °C, and 70 °C


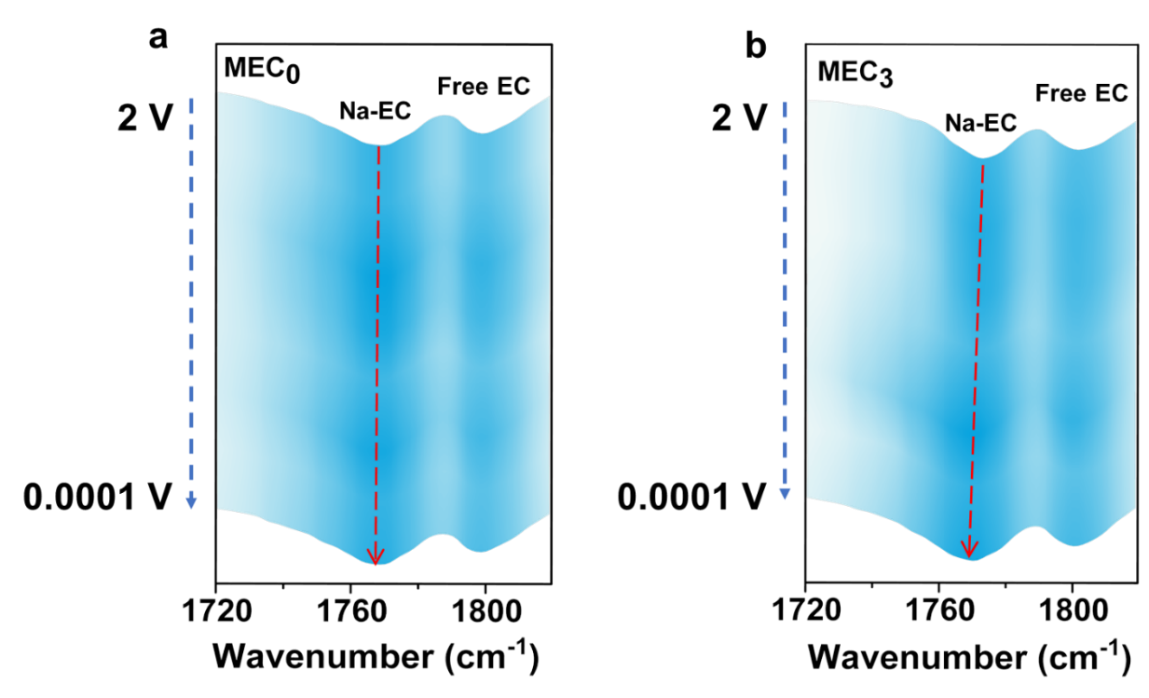


**Fig. S25** In-situ FT-IR spectra for **a** MEC_0_ and **b** MEC_3_ during the discharge process


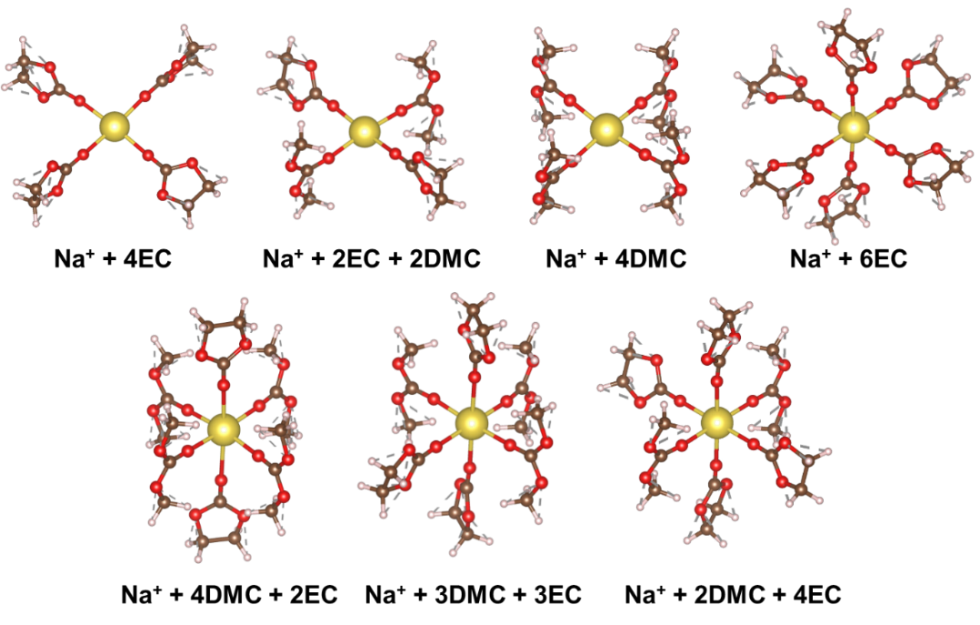


**Fig. S26** Different tetrahedral and hexahedral coordination structures of Na^+^ ions with EC, DMC, and their mixed solvents


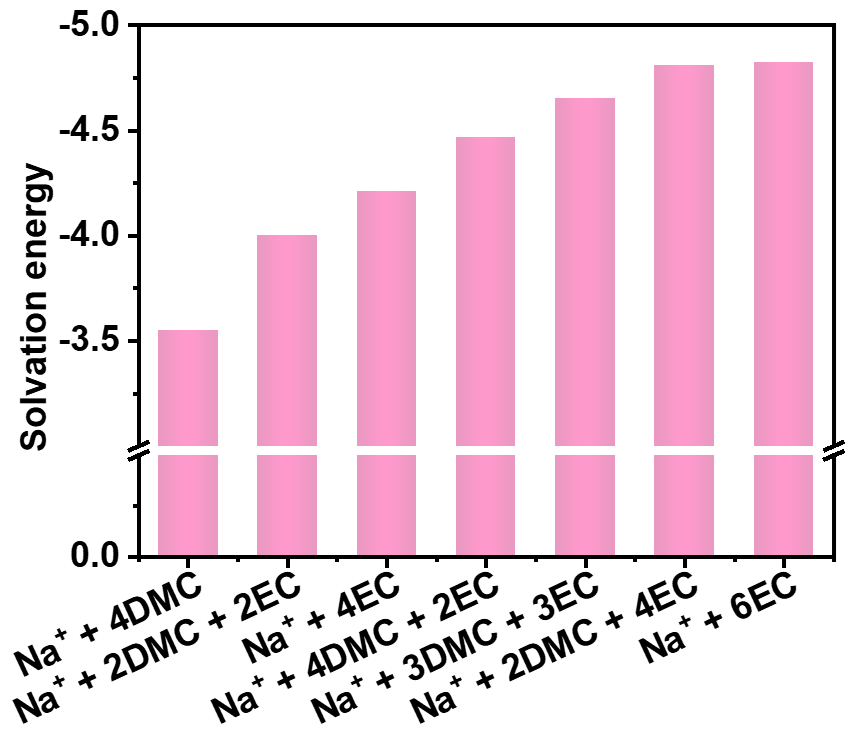


**Fig. S27** Solvation energy of different tetrahedral and hexahedral coordination structures of Na^+^ ions with EC, DMC, and their mixed solvents


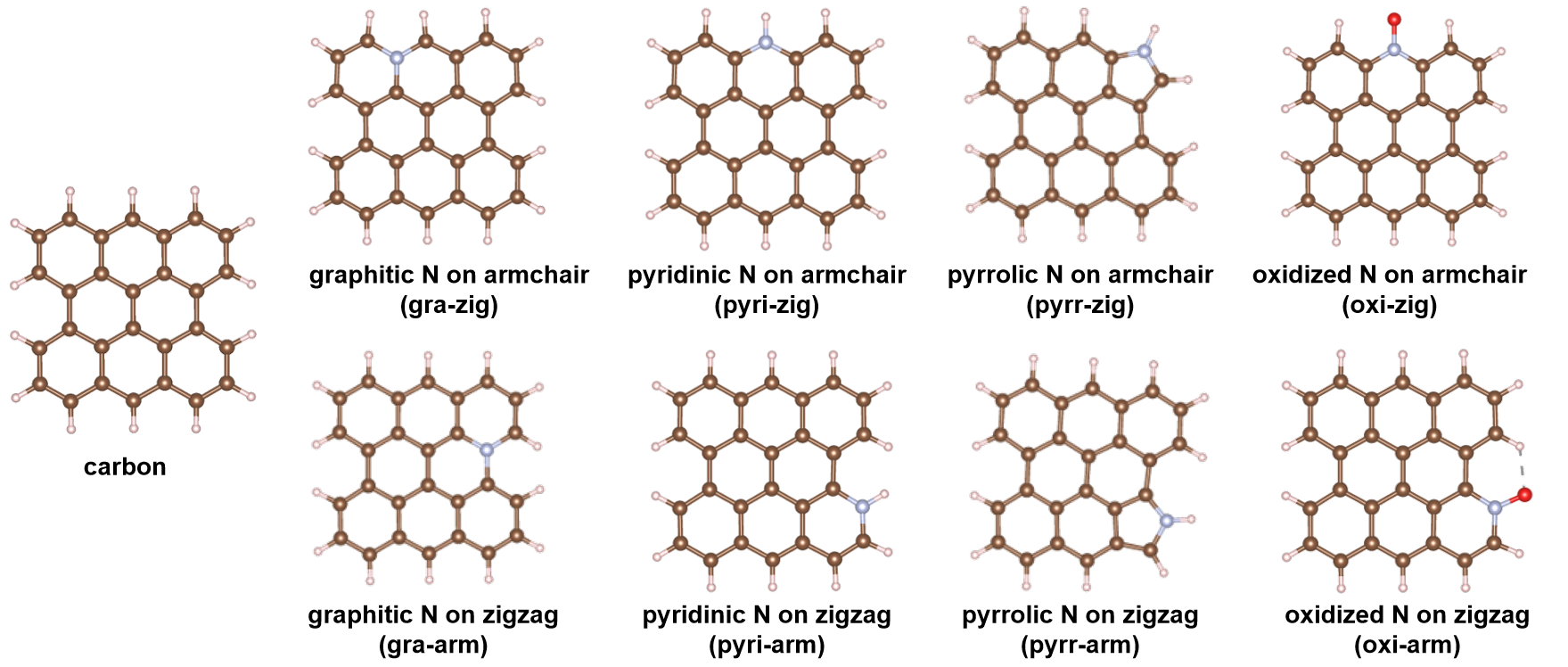


**Fig. S28** Carbon structures doped with different N species including pristine carbon, pyridinic N, pyrrolic N, graphitic N, and oxidized N featuring zigzag and armchair configurations


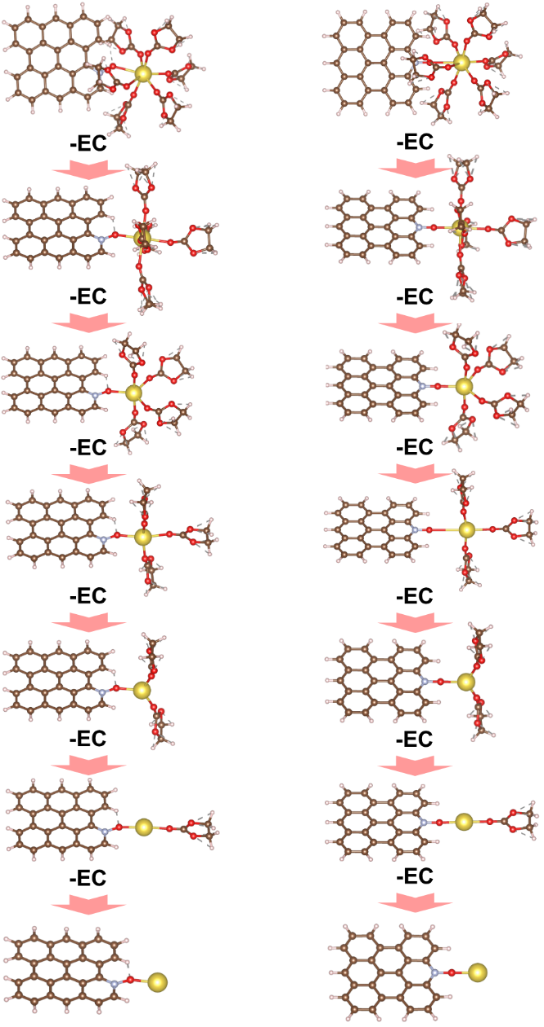


**Fig. S29** Step-wise desolvation process for the carbon structures with zigzag and armchair oxidized N


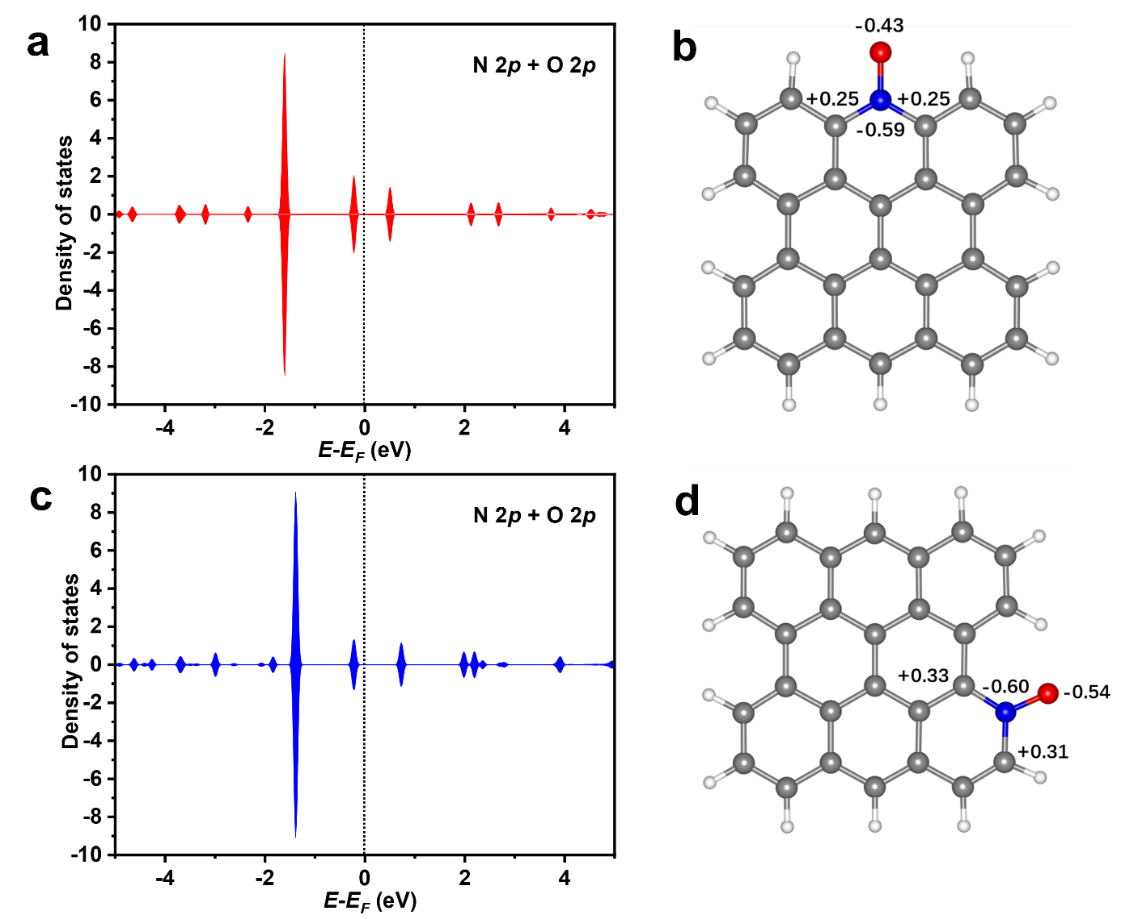


**Fig. S30** Partial density of states (pDOS) projecting on N 2*p* and O 2*p* orbitals and Bader charge analysis for oxidized N featuring **a, c** zigzag and **b, d** armchair configurations


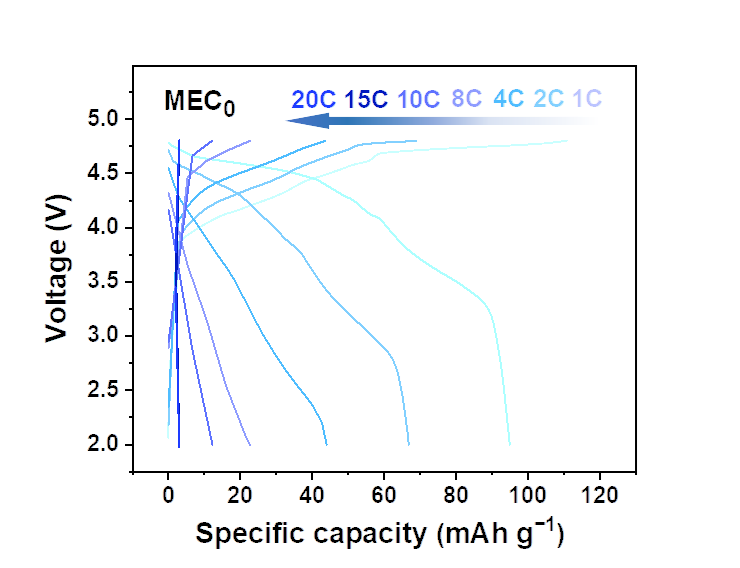


**Fig. S31** Charge/discharge curves of the MEC_0_ | | EG dual-ion battery at 1 C, 2 C, 4 C, 8 C, 10 C, 15 C, and 20 C


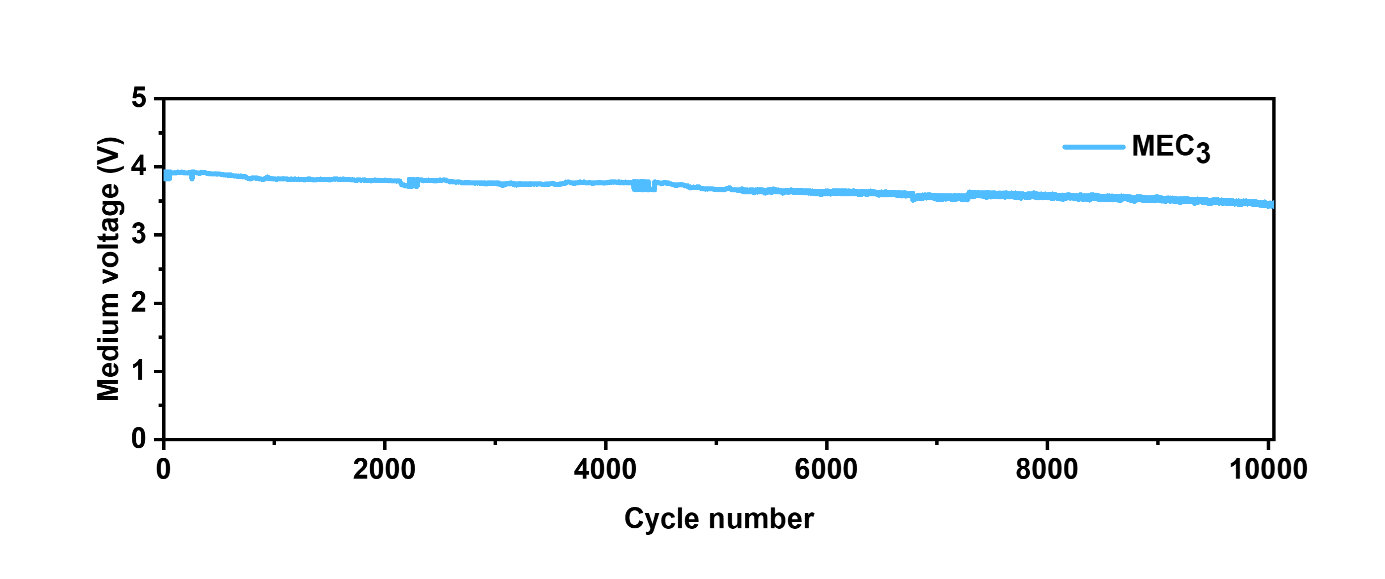


**Fig. S32** The medium voltage of The MEC_3_ | | EG cycle at 10 C

**Supplementary References**

1. F. Xie, Z. Xu, A.C.S. Jensen, F. Ding, H. Au et al., Unveiling the role of hydrothermal carbon dots as anodes in sodium-ion batteries with ultrahigh initial coulombic efficiency. J. Mater. Chem. A **7**(48), 27567–27575 (2019). <https://doi.org/10.1039/C9TA11369J>
2. Z. Xu, J. Wang, Z. Guo, F. Xie, H. Liu et al., The role of hydrothermal carbonization in sustainable sodium-ion battery anodes. Adv. Energy Mater. **12**(18), 2200208 (2022). <https://doi.org/10.1002/aenm.202200208>
3. Y. Wang, Z. Yi, L. Xie, Y. Mao, W. Ji et al., Releasing free radicals in precursor triggers the formation of closed pores in hard carbon for sodium-ion batteries. Adv. Mater. **36**(26), 2401249 (2024). <https://doi.org/10.1002/adma.202401249>
4. S. Zhang, N. Sun, X. Li, R. Ali Soomro, B. Xu, Closed pore engineering of activated carbon enabled by waste mask for superior sodium storage. Energy Storage Mater. **66**, 103183 (2024). <https://doi.org/10.1016/j.ensm.2024.103183>
5. X. Chen, N. Sawut, K. Chen, H. Li, J. Zhang et al., Filling carbon: a microstructure-engineered hard carbon for efficient alkali metal ion storage. Energy Environ. Sci. **16**(9), 4041–4053 (2023). <https://doi.org/10.1021/acsnano.4c02046>
6. J. Lin, Q. Zhou, Z. Liao, Y. Chen, Y. Liu et al., Steric hindrance engineering to modulate the closed pores formation of polymer-derived hard carbon for high-performance sodium-ion batteries. Angew. Chem. Int. Ed. **63**(39), e202409906 (2024). <https://doi.org/10.1002/anie.202409906>
7. C. Qiu, A. Li, D. Qiu, Y. Wu, Z. Jiang et al., One-step construction of closed pores enabling high plateau capacity hard carbon anodes for sodium-ion batteries: closed-pore formation and energy storage mechanisms. ACS Nano **18**(18), 11941–11954 (2024). <https://doi.org/10.1021/acsnano.4c02046>
8. Z. Lu, H. Yang, Y. Guo, H. Lin, P. Shan et al., Consummating ion desolvation in hard carbon anodes for reversible sodium storage. Nat. Commun. **15**(1), 3497 (2024). <https://doi.org/10.1038/s41467-024-47522-y>
9. W. Jian, X. Qiu, H. Chen, J. Yin, W. Yin et al., Elucidation of the sodium-ion storage behaviors in hard carbon anodes through pore architecture engineering. ACS Nano **19**(24), 22201–22216 (2025). <https://doi.org/10.1021/acsnano.5c03700>
10. J. Duan, Z. Xu, M. Li, P. Yang, H. Hu et al., Structure regulation of hard carbon with enriched semi-closed ultramicropores for enhanced rapid sodium storage. Adv. Funct. Mater. 2508822 (2025). <https://doi.org/10.1002/adfm.202508822>
11. D.-S. Bin, Y. Li, Y.-G. Sun, S.-Y. Duan, Y. Lu et al., Structural engineering of multishelled hollow carbon nanostructures for high-performance Na-ion battery anode. Adv. Energy Mater. **8**(26), 1800855 (2018). <https://doi.org/10.1002/aenm.201800855>
12. Z. Jian, Z. Xing, C. Bommier, Z. Li, X. Ji, Hard carbon microspheres: potassium-ion anode versus sodium-ion anode. Adv. Energy Mater. **6**(3), 1501874 (2016). <https://doi.org/10.1002/aenm.201501874>
13. Z. Zhu, F. Liang, Z. Zhou, X. Zeng, D. Wang et al., Expanded biomass-derived hard carbon with ultra-stable performance in sodium-ion batteries. J. Mater. Chem. A **6**(4), 1513–1522 (2018). <https://doi.org/10.1039/c7ta07951f>
14. F. Xie, Z. Xu, A.C.S. Jensen, H. Au, Y. Lu et al., Hard–soft carbon composite anodes with synergistic sodium storage performance. Adv. Funct. Mater. **29**(24), 1901072 (2019). <https://doi.org/10.1002/adfm.201901072>
15. S. Qiu, L. Xiao, M.L. Sushko, K.S. Han, Y. Shao et al., Manipulating adsorption–insertion mechanisms in nanostructured carbon materials for high-efficiency sodium ion storage. Adv. Energy Mater. **7**(17), 1700403 (2017). <https://doi.org/10.1002/aenm.201700403>
16. F. Chen, Y. Di, Q. Su, D. Xu, Y. Zhang et al., Vanadium-modified hard carbon spheres with sufficient pseudographitic domains as high-performance anode for sodium-ion batteries. Carbon Energy **5**(2), e191 (2023). <https://doi.org/10.1002/cey2.191>
17. Z. Hu, Q. Liu, K. Zhang, L. Zhou, L. Li et al., All carbon dual ion batteries. ACS Appl. Mater. Interfaces **10**(42), 35978–35983 (2018). <https://doi.org/10.1021/acsami.8b11824>
18. L. Fan, Q. Liu, S. Chen, Z. Xu, B. Lu, Soft carbon as anode for high-performance sodium-based dual ion full battery. Adv. Energy Mater. **7**(14), 1602778 (2017). <https://doi.org/10.1002/aenm.201602778>
19. X. Zhang, L. Zhang, W. Zhang, S. Xue, Y. Tang, A fast and stable sodium-based dual-ion battery achieved by Cu_3_P@P-doped carbon matrix anode. J. Power Sources **518**, 230741 (2022). <https://doi.org/10.1016/j.jpowsour.2021.230741>
20. Y. Liu, X. Hu, G. Zhong, J. Chen, H. Zhan et al., Layer-by-layer stacked nanohybrids of N, S-co-doped carbon film modified atomic MoS_2_ nanosheets for advanced sodium dual-ion batteries. J. Mater. Chem. A **7**(42), 24271–24280 (2019). <https://doi.org/10.1039/C9TA09636A>
21. M. Sheng, F. Zhang, B. Ji, X. Tong, Y. Tang, A novel tin-graphite dual-ion battery based on sodium-ion electrolyte with high energy density. Adv. Energy Mater. **7**(7), 1601963 (2017). <https://doi.org/10.1002/aenm.201601963>
22. G. Zhang, X. Ou, J. Yang, Y. Tang, Molecular coupling and self-assembly strategy toward WSe_2_/carbon micro–nano hierarchical structure for elevated sodium-ion storage. Small Meth. **5**(8), 2100374 (2021). <https://doi.org/10.1002/smtd.202100374>
23. X. Hou, W. Li, Y. Wang, S. Li, Y. Meng et al., Sodium-based dual-ion batteries *via* coupling high-capacity selenium/graphene anode with high-voltage graphite cathode. Chin. Chem. Lett. **31**(9), 2314–2318 (2020). <https://doi.org/10.1016/j.cclet.2020.04.021>
24. H. Wang, Q. Wu, Y. Wang, X. Lv, H.-G. Wang, A redox-active metal–organic compound for lithium/sodium-based dual-ion batteries. J. Colloid Interface Sci. **606**, 1024–1030 (2022). <https://doi.org/10.1016/j.jcis.2021.08.113>
25. S. Mu, Q. Liu, P. Kidkhunthod, X. Zhou, W. Wang et al., Molecular grafting towards high-fraction active nanodots implanted in N-doped carbon for sodium dual-ion batteries. Natl. Sci. Rev. **8**(7), nwaa178 (2020). <https://doi.org/10.1093/nsr/nwaa178>
26. Z. Guo, Z. Xu, F. Xie, J. Jiang, K. Zheng et al., Investigating the superior performance of hard carbon anodes in sodium-ion compared with lithium- and potassium-ion batteries. Adv. Mater. **35**(42), 2304091 (2023). <https://doi.org/10.1002/adma.202304091>
27. N. Jiang, L. Chen, H. Jiang, Y. Hu, C. Li, Introducing the solvent co-intercalation mechanism for hard carbon with ultrafast sodium storage. Small **18**(15), 2108092 (2022). <https://doi.org/10.1002/smll.202108092>
28. W. Li, X. Guo, K. Song, J. Chen, J. Zhang et al., Binder-induced ultrathin SEI for defect-passivated hard carbon enables highly reversible sodium-ion storage. Adv. Energy Mater. **13**(22), 2300648 (2023). <https://doi.org/10.1002/aenm.202300648>
29. Z. Wen, R. Zhao, T. Tian, T. Zhang, X. Wang et al., Molecular stitching in polysaccharide precursor for fabricating hard carbon with ultra-high plateau capacity of sodium storage. Adv. Mater. **37**(18), 2420251 (2025). <https://doi.org/10.1002/adma.202420251>
